# *Aedes aegypti* Infection With Trypanosomatid *Strigomonas culicis* Alters Midgut Redox Metabolism and Reduces Mosquito Reproductive Fitness

**DOI:** 10.3389/fcimb.2021.732925

**Published:** 2021-08-13

**Authors:** Ana Cristina S. Bombaça, Ana Caroline P. Gandara, Vitor Ennes-Vidal, Vanessa Bottino-Rojas, Felipe A. Dias, Luana C. Farnesi, Marcos H. Sorgine, Ana Cristina Bahia, Rafaela V. Bruno, Rubem F. S. Menna-Barreto

**Affiliations:** ^1^Laboratório de Biologia Celular, Instituto Oswaldo Cruz, Fiocruz, Rio de Janeiro, Brazil; ^2^Laboratório de Bioquímica de Artrópodes Hematófagos, Instituto de Bioquímica Médica, Universidade Federal do Rio de Janeiro, Rio de Janeiro, Brazil; ^3^Laboratório de Estudos Integrados em Protozoologia, Instituto Oswaldo Cruz, Fiocruz, Rio de Janeiro, Brazil; ^4^Laboratório de Biologia Molecular de Insetos, Instituto Oswaldo Cruz, Fiocruz, Rio de Janeiro, Brazil; ^5^Laboratório de Bioquímica de Insetos e Parasitos, Instituto de Biofísica Carlos Chagas Filho, Universidade Federal do Rio de Janeiro, Rio de Janeiro, Brazil; ^6^Instituto Nacional de Ciência e Tecnologia em Entomologia Molecular (INCT-EM/CNPq), Rio de Janeiro, Brazil

**Keywords:** *Aedes aegypti*, *Strigomonas culicis*, dual oxidase, catalase, reactive oxygen species, fecundity, fertility

## Abstract

*Aedes aegypti* mosquitoes transmit arboviruses of important global health impact, and their intestinal microbiota can influence vector competence by stimulating the innate immune system. Midgut epithelial cells also produce toxic reactive oxygen species (ROS) by dual oxidases (DUOXs) that are essential players in insect immunity. *Strigomonas culicis* is a monoxenous trypanosomatid that naturally inhabits mosquitoes; it hosts an endosymbiotic bacterium that completes essential biosynthetic pathways of the parasite and influences its oxidative metabolism. Our group previously showed that *S. culicis* hydrogen peroxide (H_2_O_2_)-resistant (WTR) strain is more infectious to *A. aegypti* mosquitoes than the wild-type (WT) strain. Here, we investigated the influence of both strains on the midgut oxidative environment and the effect of infection on mosquito fitness and immunity. WT stimulated the production of superoxide by mitochondrial metabolism of midgut epithelial cells after 4 days post-infection, while WTR exacerbated H_2_O_2_ production mediated by increased DUOX activity and impairment of antioxidant system. The infection with both strains also disrupted the fecundity and fertility of the females, with a greater impact on reproductive fitness of WTR-infected mosquitoes. The presence of these parasites induced specific transcriptional modulation of immune-related genes, such as *attacin* and *defensin A* during WTR infection (11.8- and 6.4-fold, respectively) and *defensin C* in WT infection (7.1-fold). Thus, we propose that *A. aegypti* oxidative response starts in early infection time and does not affect the survival of the H_2_O_2_-resistant strain, which has a more efficient antioxidant system. Our data provide new biological aspects of *A. aegypti*–*S. culicis* relationship that can be used later in alternative vector control strategies.

## Introduction

*Aedes aegypti* is the primary vector of arboviruses, such as dengue, Zika, chikungunya, and urban yellow fever, which cause a heavy health burden and global economic losses (Lwande et al., 2020). The distribution of this vector and consequent transmission of these pathogens were for a long time restricted to particular regions. This scenario has changed, and today, it colonizes almost all continents and is widespread in tropical and subtropical areas (Kraemer et al., 2019). *A. aegypti* is considered an extremely efficient vector due to its anthropophilic nature and the preference for living around or inside human dwellings (Lwande et al., 2020).

Although mosquito females are facultative hematophagous, they require the vertebrate blood meal for egg production (Attardo et al., 2005). The digestion of hemoglobin, the most abundant protein of mammalian blood, releases large amounts of amino acids and peptides, which are used by the mosquito for energy production. Hemoglobin digestion is also responsible for the exposure of the insects’ midgut to a heme overload, a pro-oxidant molecule able to generate high levels of reactive oxygen species (ROS) (Graça-Souza et al., 2006; Sterkel et al., 2017). NADPH oxidases are important ROS producers in midgut epithelial cells of insects, more specifically dual oxidase (DUOX). This enzyme is responsible for generating hydrogen peroxide (H_2_O_2_) in response to a wide range of stimuli, being involved in diverse aspects of insects’ gut–microbe interactions (Kim and Lee, 2014). DUOX possesses two main regulatory pathways: a DUOX–activity pathway, which causes intracellular Ca^2+^ release to regulate enzymatic activity, and a DUOX–expression pathway, which regulates its gene expression. The activation of both regulatory pathways is necessary for the robust pro-oxidative response (Bai et al., 2021).

Previous studies reported that ROS production plays a key role in the insect innate immune responses, being a potent pathogen-killing agent as demonstrated in *Plasmodium*-infected mosquitoes. In *Anopheles gambiae*, the infective refractory profile was related to the chronic production of ROS and oxidative stress exacerbation after blood feeding. Catalase-silenced mosquitoes fed with *Plasmodium berghei*-infected meal also produced higher levels of H_2_O_2_ that favored parasite clearance through a lytic mechanism (Kumar et al., 2003; Molina-Cruz et al., 2008). Heme ingestion and DUOX-silencing decreased ROS levels in a protein kinase C (PKC)-dependent mechanism and increased proliferation of endogenous bacteria in *A. aegypti* (Oliveira et al., 2011). The mosquito shuts down ROS production and induces catalase expression after a blood meal as a strategy to avoid heme-mediated oxidative stress in the gut, evidencing the cytotoxic effects of uncontrolled ROS production. Interestingly, this protection facilitates the establishment of Dengue virus in the midgut (Oliveira et al., 2017). A similar mechanism was also observed in *Drosophila melanogaster*, where an immune-regulated catalase, an enzyme that catalyzes the conversion of H_2_O_2_ into H_2_O, is overexpressed to regulate the oxidative balance (Ha et al., 2005).

Trypanosomatidae family (Euglenozoa: Kinetoplastida) is a diverse group of protist parasites, widely known for sheltering etiological agents of important human diseases. Many trypanosomatids are predominantly monoxenous, undergoing complete development in a single invertebrate host, and are generally considered non-pathogenic. The current data on monoxenous parasites’ development point to Diptera and Hemiptera as the prevalent orders to host these trypanosomatids, acquiring them through predation, necrophagy, cannibalism, and coprophagy behavior (Frolov et al., 2021). *Strigomonas culicis* is a promiscuous monoxenous trypanosomatid isolated from several mosquito species (Wallace, 1966). Artificial infection of *A. aegypti* by *S. culicis* showed that the parasites remained for a long time in the insect digestive tract, being able to induce midgut cell degradation and hemocoel penetration. This trypanosomatid was also able to inhabit the mosquito hemolymph, invading the host salivary gland (Corrêa-Da-Silva et al., 2006; Nascimento et al., 2010).

*S. culicis* is largely recognized to bear a single obligate bacterium in its cytoplasm, named *Candidatus* Kineto- plastibacterium blastocrithidii (Teixeira et al., 2011). Biochemical studies revealed that the endosymbiont completes essential metabolic pathways of the host parasite, such as amino acid production and heme biosynthesis (Chang et al., 1975; Alves et al., 2011; Alves et al., 2013). In this obligatory association, the bacterium is unable to survive and replicate out of the host, whereas the aposymbiotic strain (chloramphenicol-treated parasites) is incapable of colonizing insects (Fampa et al., 2003). Previous work of our group demonstrated that symbiont elimination generated an intense susceptibility to oxidative stress in the aposymbiotic strain. Results obtained after feeding the mosquitoes with a sodium L-ascorbate (ASC)-enriched diet showed that in this condition, the aposymbiotic strain reaches infectivity rates similar to wild-type (WT) strain and remains in the mosquito midgut for a long time. As a counterpart, with wild-type H_2_O_2_-resistant (WTR) strain, a large infection in the mosquito’s midgut was observed, suggesting that reactive species can control *S. culicis* population during invertebrate colonization (Bombaça et al., 2017). Considering that many monoxenous trypanosomatids influence their host population (Kostygov et al., 2021), in the present study, we analyzed the impact of *S. culicis* infection in *A. aegypti* reproductive fitness, also examining, for the first time, the influence of monoxenous parasites on egg laying and development. We also evaluated the contribution of classical immune-related enzymes, as DUOX and CAT, to the establishment of trypanosomatid infection in insects’ midgut and Aag2 cell culture, especially by H_2_O_2_-resistant strain.

## Materials and Methods

### Parasites

*S. culicis* epimastigotes were maintained at 28°C in liver infusion and tryptose (LIT) medium, supplemented with 10% heat-inactivated fetal bovine serum (FBS; Cultilab, Campinas, Brazil). Briefly, WT, aposymbiotic (Apo), and WTR strains were counted every 4 days and added to a fresh medium at a final concentration of 3 × 10^6^ parasites/ml. All *A. aegypti* infections were performed with 3-day-old culture epimastigotes, which correspond to parasite exponential growth phase. In addition, for the resistance induction, WT strain was successively cultivated with increasing concentrations of H_2_O_2_ (Merck, Darmstadt, Germany) up to 1 mM (Bombaça et al., 2017).

### Mosquito Maintenance and *S. culicis* Infection

*A. aegypti* (Red Eye strain) was reared in insectaries at Universidade Federal do Rio de Janeiro or at Instituto Oswaldo Cruz, Brazil, under a 12-h light/dark cycle at 25°C ± 2°C and 40%–80% relative humidity. Briefly, 300 mosquito larvae were fed with fish food (TetraMin Tablets; Tetra, Melle, Germany) every 2 days until pupae development, while adult mosquitoes (males and females) were kept in cages and fed with a solution of 10% sucrose *ad libitum* (Farnesi et al., 2009). Here, 4–7-day-old females were starved for up to 12 h and then artificially fed for 1 h with *S. culicis* suspension (10^8^ parasites/ml in a solution of 10% sucrose + 1 mM ATP) using water-jacketed artificial feeders maintained at 37°C and sealed with parafilm membranes (Bombaça et al., 2017). Assays were performed in female mosquitoes 4 days post-infection (dpi) and under three different *ad libitum* diet conditions: (i) 10% sucrose, (ii) 10% sucrose + 5 mM ASC (Sigma-Aldrich, St. Louis, USA), or (iii) 10% sucrose + 10 µM diphenylene iodonium (DPI; Sigma-Aldrich) solutions.

### Oviposition Efficiency and Egg Viability

For fecundity and fertility assays, inseminated females (5 days old) were collected and infected with the three *S. culicis* strains as described above. On the next day, blood meals required for egg production were allowed for 30–60 min in anesthetized *Swiss* mice (CEUA/FIOCRUZ LW-20/14), and engorged females were collected to continue the experiment. After 3 days, the synchronized oviposition was stimulated through the distribution of individual mosquitoes in Petri dishes (150 mm diameter, lined with filter paper) and incubation with 4 ml filtered water for 1 h in the dark. The females were then discarded, and the number of eggs per female was counted (Rezende et al., 2008; Vargas et al., 2014). For egg viability, 50 ml of 0.15% yeast solution (w/v) was added in each Petri dish and maintained for 24 h at 25°C ± 2°C and 40%–80% relative humidity. After that, the hatching of L1 larvae was calculated (Farnesi et al., 2009). The parameters analyzed were (a) the percentage of efficient females (egg production equal to or greater than the median production of control mosquitoes), (b) null females (egg production equal to zero), and (c) infertile females (egg hatching equal to zero) (Resck et al., 2020).

### High-Performance Liquid Chromatography (HPLC) Assessment of Dihydroethidium (DHE) Products and Fluorescence Microscopy

To analyze the ROS amounts produced during *A. aegypti* infection, midguts were dissected and the oxidation products of DHE (Invitrogen, Carlsbad, USA) were assessed (Fernandes et al., 2007). Briefly, midguts were opened and washed with 10 mM phosphate buffer saline (PBS) to remove the intestinal contents. After that, pools of 15 gut epithelia each were incubated with 100 µM DHE for 30 min at 28°C, washed twice with PBS, frozen in liquid N_2_, and homogenized in 100% acetonitrile (500 µl). The samples were sonicated and centrifuged at 13,000 *g* for 10 min; the resulting supernatant was dried under vacuum, and the pellet obtained was stored at -70°C until use. The dried pellets were resuspended in PBS containing 100 µM diethylenetriaminepentaacetic acid (DTPA; Sigma-Aldrich) and injected into a HPLC LC-10AT device (Shimadzu, Kyoto, Japan) equipped with a diode array (SPD-M10A) and fluorescence (RF-20A) detectors. Chromatographic separation of DHE oxidation products was performed using NovaPak C18 column, with 4 µM particle size and 3.9 mm × 150 mm (Waters, Milford, USA), equilibrated in solution A (10% acetonitrile and 0.1% trifluoroacetic acid ) at a flow rate of 0.4 ml/min. After sample injection, a 0%–40% linear gradient of solution B (100% acetonitrile) was applied for 10 min, followed by 10 min of 40% solution B, 5 min of 100% solution B, and 10 min of 100% solution A. The amount of DHE was measured by light absorption at 245 nm, and the DHE oxidation products, hydroxyethidium (EOH) and ethidium (E), were monitored by fluorescence detection with excitation at 510 nm and emission at 595 nm. For fluorescence microscopy analysis, *A. aegypti* midguts were dissected and incubated with 50 µM DHE as described above. After the incubation period, midguts were washed twice, fixed in 4% paraformaldehyde (Sigma-Aldrich) for 30 min at room temperature, and transferred for a glass slide. Semiquantitative evaluation of fluorescence levels was performed by acquiring three images under identical conditions of each midgut using a ×40 objective and 100 ms exposure time. The images were acquired by a Zeiss AxioObserver M1 microscope (Carl Zeiss, Oberkochen, Germany), and the data were analyzed using the ImageJ software (Bethesda, MD, USA). Representative sections of midguts of each experimental condition were analyzed under an LSM 710 confocal microscope (Carl Zeiss, Germany) at Plataforma de Microscopia Confocal (Instituto Oswaldo Cruz, Brazil).

### Cell Culture and *S. culicis* Infection

*A. aegypti* Aag2 cells (Grace, 1966) were maintained at 28°C in Schneider’s Insect Medium (Sigma-Aldrich) supplemented with L-glutamine and 10% FBS. Cultures with 3-days-old (80%–90% confluence) were enzymatically dissociated with 1% trypsin (Sigma-Aldrich) and plated for 72 h to establish a cellular monolayer. After that, cells were quantified and infected with WT and WTR strains [multiplicity of infection (MOI) 1:1] for 4 h, washed with PBS to remove the non-adhered parasite, and maintained until 24 h at 28°C. The culture was then stained with fast panoptic (Laborclin, Pinhais, Brazil), and the percentage of cells with adhered parasite and the number of parasites per 100 cells were calculated. Alternatively, cells were treated with different pro-oxidant or antioxidant agents for 2 h before *S. culicis* infection. Treatments with 0.25 µM DPI, 5 µM menadione (Sigma-Aldrich), 50 U/ml superoxide dismutase (SOD; Sigma-Aldrich) from bovine erythrocytes, 50 U/ml catalase (Sigma-Aldrich) from bovine liver, and 1 µM mitoTEMPO (Santa Cruz Biotechnology, Dallas, USA) were maintained up to 24 h.

### Hydrogen Peroxide Release and Dual Oxidase Activity

To evaluate the H_2_O_2_ production, infected midguts were dissected, and the intestinal contents were washed out with PBS. Pools of 10–20 gut epithelia were incubated in 100 µl PBS under dim light and at room temperature with 100 µM Amplex Red reagent (Invitrogen) and 50 U/ml horseradish peroxidase (HRP; Sigma-Aldrich) for 30 min. After this period, the midguts were spun, and the supernatant was analyzed to H_2_O_2_ presence. Since DUOX activity depends on calcium presence to H_2_O_2_ generation, the evaluation was made before the pre-incubation of midguts (pools of 15 epithelia each) in 200 µM Schneider’s Insect Medium with 1 µM ionomycin (Iono; Sigma-Aldrich), 5 mM ethylene glycol bis(2-aminoethyl ether)-N,N,N’,N’-tetraacetic acid (EGTA; Sigma-Aldrich), and 5 µM DPI for 10 min at room temperature. After that, samples were incubated with Amplex Red and HRP for 60 min, and the supernatant was assessed for H_2_O_2_ release (Dias et al., 2013). Resorufin fluorescence was monitored by a SpectraMax M3 fluorimeter (Molecular Devices, Sunnyvale, USA) with excitation at 530 nm and emission at 590 nm and compared to H_2_O_2_ standard curves. Infected Aag2 cells were also evaluated for H_2_O_2_ production and DUOX activity. Thus, the cells were submitted to the abovementioned protocol, and the supernatants (100 µl) were used for fluorescence assessment. After that, the cellular culture was washed twice with PBS, and the cells were mechanically dissociated. Homogenates were obtained by sonication, and the protein concentration was assessed by Pierce™ BCA protein assay kit (Thermo Fisher Scientific, Waltham, USA) according to the manufacturer’s instructions.

### Antioxidant and Mitochondrial Enzymes Activities

Pools of 15 midguts were used for catalase and citrate synthase (CS) activity assessment. Briefly, the guts were dissected, washed out for content elimination, and disrupted by sonication for protein determination. In parallel, infected Aag2 cells were processed as described above. For catalase activity measurement, the homogenates were added to the reaction mixture containing 100 mM potassium phosphate buffer and 100 µM H_2_O_2_ (Weydert and Cullen, 2010). Peroxide disappearance was monitored for 2 min and recorded every 30 s at 240 nm, and positive control was made with 4 U/ml catalase. CS was measured through the reduction of coenzyme A using the thiol reagent 5,5′-dithiobis(2-nitrobenzoic acid) (DTNB) at 412 nm. The reaction mixture consisted of 75 mM Tris-HCl buffer (pH 8.0), with 30 µM acetyl-CoA (Sigma-Aldrich), 250 µM DTNB, and 500 µM oxaloacetate (Sigma-Aldrich) to start the reaction. Aag2 homogenates were also evaluated for mitochondrial complex I–III activity through ferricytochrome *c* reduction at 550 nm. Briefly, the samples were added to 50 mM potassium phosphate buffer (pH 7.5) with 1 mg/ml bovine serum albumin (BSA), 300 µM potassium cyanide (KCN; Merck, Darmstadt, Germany), 50 µM equine heart cytochrome *c* (Sigma-Aldrich), and 200 µM β-Nicotinamide adenine dinucleotide (NADH; Sigma-Aldrich). All assays were performed at room temperature in a SpectraMax M3 spectrometer and calculated by molar extinction coefficient of H_2_O_2_ (ε = 39.4 mmol^−1^cm^−1^), DTNB (ε = 13.6 mmol^-1^cm^-1^), and cytochrome c (ε = 18.5mmol^−1^cm^−1^) (Spinazzi et al., 2012).

### Ultrastructural Analysis

Aag2 cells infected or not with *S. culicis* strains were fixed with 2.5% glutaraldehyde (Sigma-Aldrich) diluted in 0.1 M Na-cacodylate buffer (pH 7.2) for 40 min and postfixed with 1% osmium tetroxide (Sigma-Aldrich) with 2.5 mM calcium chloride and 0.8% potassium ferricyanide for 20 min, both at room temperature. After the washings, dehydration was performed in ethanol (50%, 70%, 90%, and 100%), dried by the critical point method with CO_2_, mounted on aluminum stubs, coated with a 20-nm-thick gold layer, and analyzed in Jeol JSM6390LV (Jeol Ltd., Tokyo, Japan) scanning electron microscope, located in Plataforma de Microscopia Eletrônica Rudolf Barth (Instituto Oswaldo Cruz, Brazil).

### Quantitative PCR (qPCR) Analysis

For gene expression analysis, total RNA was isolated from 10–20 midguts using TRIzol reagent (Thermo Fisher Scientific) according to the manufacturer’s protocol. Complementary DNA was synthesized with SuperScript Vilo kit (Invitrogen) using oligo(dT) primers. All primers were tested by conventional PCR using a 40-cycle reaction (denaturation at 95°C for 30 s, annealing at 60°C for 30 s, and extension at 72°C for 30 s), and PCR products were separated in 2% agarose gel. The qPCR was performed in an ABI Prism 7500 FAST (Applied Biosystems, Foster City, CA, USA) using ~40 ng/μl cDNA, specific primers, and Go-Taq PCR Master Mix (Promega, Madison, WI, USA). The comparative ΔΔCt method was used to compare changes in gene expression levels (Livak and Schmittgen, 2001). Genes of ribosomal protein S7 and glyceraldehyde 3-phosphate dehydrogenase (GAPDH) were used as reference genes of *A. aegypti* and *S. culicis*, respectively. All oligonucleotide sequences used in qPCR assays are listed in **Table S1**.

### Statistical Analysis

Analyses were performed with GraphPad Prism version 5.0 for Windows (GraphPad Software, San Diego, USA) or IBM SPSS Statistics 22.0 software (IBM Corporation, New York, NY, USA). Asterisks indicate significant differences with the threshold for significance set at p ≤ 0.05. The pairwise comparisons and each used posttest analysis are described in respective figure legends.

## Results

### *S. culicis* Triggers Different Oxidative Responses During *A. aegypti* Infection

**Figure 1:**
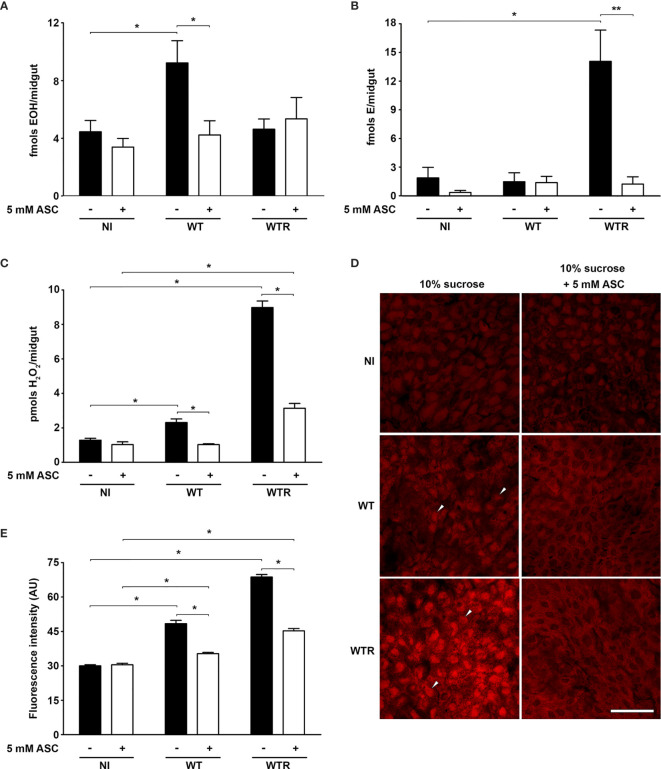
*Strigomonas culicis* infection in midgut elicited a reactive oxygen species (ROS)-dependent response in *Aedes aegypti*. Analysis was performed in midguts of non-infected (NI), wild-type (WT) and hydrogen peroxide (H_2_O_2_)-resistant (WTR)- infected females fed with 10% sucrose or 10% sucrose + 5 mM sodim L-ascorbate (ASC) *ad libitum* 4 dpi. High-performance liquid chromatography (HPLC) analysis of dihydroethidium (DHE) oxidation products **(A)** hydroxyethidium (EOH) (*p = 0.02) and **(B)** ethidium E (*p = 0.03 and **p = 0.02) and **(C)** total H_2_O_2_ production measurement (*p = 0.05). Assays were performed in technical replicate with pools of 10–15 guts. **(D)** Representative confocal images of midguts dissected and incubated with DHE. Scale bar represents 40 µm. Arrowheads indicate high staining in the infected midgut. **(E)** Quantitative analysis of fluorescence microscopy (*p = 0.001). Individual analysis of 10–15 midguts per group. Significant p-values were obtained by Mann–Whitney test, and error bars represent the mean ± SEM of at least three independent experiments.

As previous data pointed out that an antioxidant-enriched diet increases *S. culicis* infection in *A. aegypti* females (Bombaça et al., 2017), we decided to evaluate the contribution of ROS in response to infection. DHE oxidation analysis and H_2_O_2_ quantification were performed to assess the redox state of infected midguts from sucrose- or ASC-fed females. Primarily, as DHE oxidation is known to yield two distinct products according to oxidative species available, the concentration of EOH (selectively formed when DHE is oxidized by superoxide) and E (generated through reactions with different oxidant species) (Zhao et al., 2003) in tissues 4 dpi was differentially assessed through HPLC. WT infection increases the EOH levels 2.1-fold in sugar-fed mosquitoes, while no significant difference was detected in E amount. In contrast, WTR infection exacerbated only the production of E, increasing its detection 9.7-fold in infected midguts. The antioxidant-enriched diet prevented ROS production in infected tissues and conserved the EOH and E levels as in the diet-matched control group (**Figures 1A, B**). To further confirm that non-superoxide oxygen species are increased in WTR-infected mosquitoes, H_2_O_2_ release in midguts was measured. Our data pointed out that H_2_O_2_ was produced during *S. culicis* infection, increasing detection 1.8- and 7.0-fold after WT and WTR infections, respectively. Once again, the antioxidant-enriched diet limited ROS generation upon infection, decreasing up to 65.0% the H_2_O_2_ release compared to sugar-fed infected mosquitoes (**Figure 1C**).

Midguts of non-infected (NI) and infected mosquitoes were also incubated with DHE to determine the oxidative state by fluorescence microscopy. Infected midguts showed a clear increase in DHE staining comparing to NI controls, especially after WTR infection. These data reflect the increase of 1.6- and 2.3-fold in fluorescence intensity observed after the infection with WT and WTR, respectively. Microscopy analysis also allowed the detection of the effects of diet supplementation. ASC-fed mosquitoes presented that ROS production in response to *S. culicis* infection was reduced up to 70% (**Figures 1D, E**). Our microscopy findings also reflected the general observations about oxidative state of infected midguts, being corroborated by the analysis of DHE oxidation products (**Figures 1A, B, E**). To confirm that ROS production is a direct immunological response against *S. culicis*, DHE fluorescence analysis was performed in midguts infected with Apo strain, given that ASC feeding is able to modulate protist infection in the midguts (Bombaça et al., 2017). Fluorescence intensity was greater in infected midguts (up to 5.4-fold) 1 dpi regardless of antioxidant feeding. This profile changes 4 dpi in sugar-fed insects, when similar fluorescence detection was observed between NI and infected mosquitoes. However, at 4 dpi, fluorescence intensity was 2.9-fold higher in Apo-infected ASC-fed group compared to diet-matched NI mosquitoes, demonstrating a time-dependent increase of ROS production upon infection (**Figure S1**).

### Dual Oxidase Produces High Hydrogen Peroxide Levels in Response to WTR Infection in Midgut Epithelial Cells

**Figure 2:**
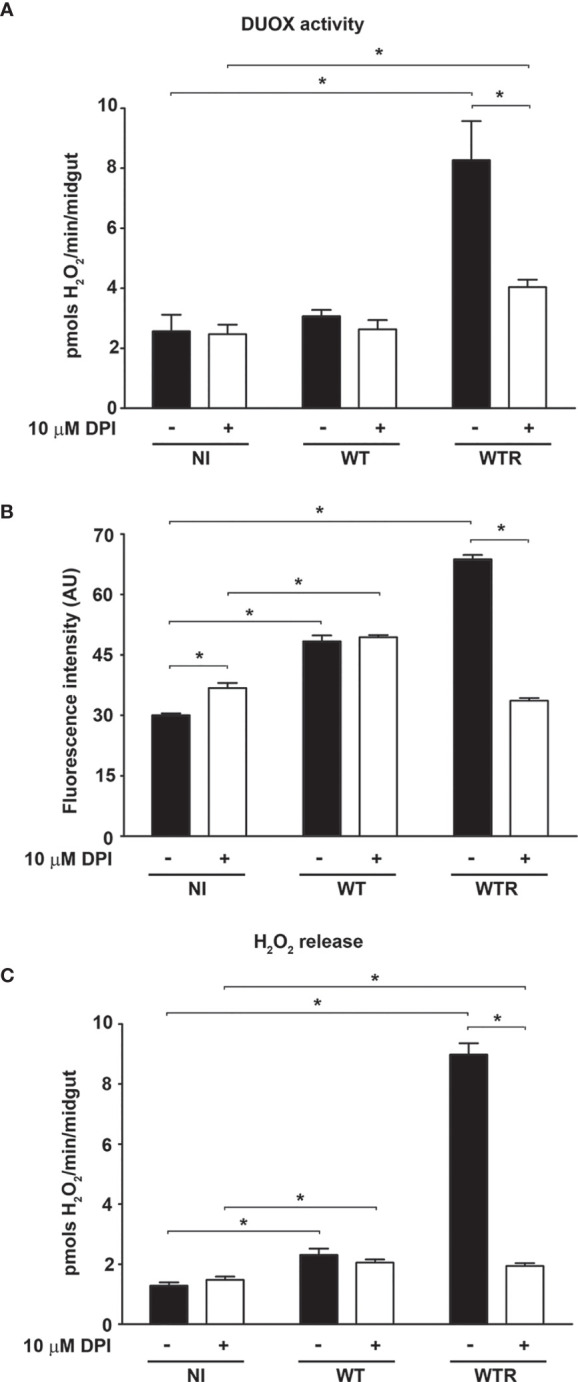
*Strigomonas culicis* hydrogen peroxide (H_2_O_2_)-resistant (WTR) strain stimulates dual oxidase (DUOX)-dependent H_2_O_2_ production during *Aedes aegypti* infection. Analysis was performed in midguts of non-infected (NI), wild-type (WT)- and WTR-infected females fed with 10% sucrose or 10% sucrose + 10 µM diphenylene iodonium (DPI) *ad libitum* 4 dpi. **(A)** Ionomycin-derived DUOX activity (*p = 0.05). Enzyme activity was performed through the production of H_2_O_2_ in midguts incubated with 1 µM ionomycin (pools of 20 guts). **(B)** Quantitative analysis of fluorescence microscopy (*p = 0.001). Individual analysis of 10–15 midguts per group. **(C)** Total H_2_O_2_ production measurement (*p = 0.05). Significant p-values were obtained by Mann–Whitney test, and error bars represent the mean ± SEM of at least three independent experiments.

**Figure 3:**
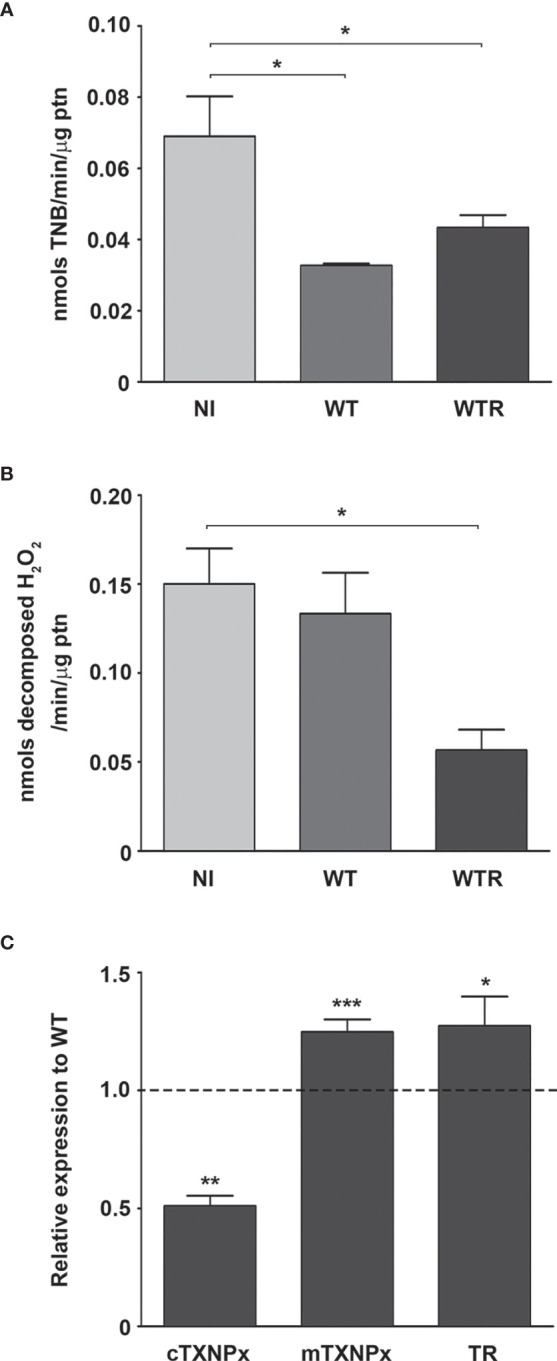
Citrate synthase (CS) and catalase activities of *Aedes aegypti* were downregulated by the infection with Strigomonas culicis wild-type (WT) and hydrogen peroxide (H_2_O_2_)-resistant (WTR) strains, respectively. **(A)** CS and **(B)** catalase activities in midguts of non-infected (NI) and WT- and WTR-infected females fed with 10% sucrose *ad libitum* 4 dpi (*p = 0.05). Activities were performed in pools of 15 guts. **(C)** Transcript levels of cytosolic tryparedoxin peroxidase (cTXNPx), mitochondrial tryparedoxin peroxidase (mTXNPx), and trypanothione reductase (TR) of WTR during midgut infection (*p = 0.01, **p = 0.007, ***p = 0.003). Gene expression was normalized using the transcript levels of WT strain (dashed line). Glyceraldehyde 3-phosphate dehydrogenase (GAPDH) was used as the endogenous control. Significant p-values were obtained by Mann–Whitney test, and error bars represent the mean ± SEM of three independent experiments.

To investigate the mechanisms involved in strain-specific response produced by *A. aegypti*, the DUOX activity in infected midguts was analyzed. DUOX enzymes are calcium-dependent NADPH oxidases, and the incubation of NI midguts with ionomycin markedly stimulated H_2_O_2_ production. Additionally, the incubation of tissues with DPI, an inhibitor of flavoenzymes, or EGTA, a calcium-chelating agent, prevented the ionomycin-induced increase in H_2_O_2_ production up to 80% (**Figure S2A**). The contribution of DUOX to the oxidative burst during *S. culicis* infection was confirmed by analyses of enzyme activity and ROS production and the addition of 10 µM DPI into mosquitoes’ diet. First, we observed an increase of 3.2-fold in DUOX activity in WTR-infected mosquitoes, while DPI feeding decreased it significantly, in response to *S. culicis* infection (**Figure 2A**). DHE and Amplex Red assays evidenced that ROS generation in infected midguts was prevented by DPI, decreasing the fluorescence intensity 51.0% and the H_2_O_2_ release 78.3% after WTR infection (**Figures 2B, C** and **Figure S2B**). As our results demonstrated that WT infection did not change DUOX activity, and oxidative stress remained high even in the DPI-fed group (**Figure 2**), the mitochondrial activity of epithelial midgut cells was analyzed. The evaluation of CS activity, a mitochondrial content marker enzyme (Spinazzi et al., 2012), revealed an impairment of 52.5% and 37.3% after WT and WTR infections, respectively (**Figure 3A**). Since the balance between ROS-generating systems and ROS-detoxifying reactions is critical for oxidative homeostasis (Sterkel et al., 2017), the antioxidant state of *A. aegypti* midguts and *S. culicis* strains during infection was assessed. WTR-infected midguts showed a decrease of 63.5% catalase activity, while no significant differences were detected after WT infection (**Figure 3B**). As a counterpart, qPCR analysis presented a modulation of the parasite’s antioxidant gene expression. In relation to WT strain, WTR showed an increment of up to 1.3-fold in trypanothione reductase (TR) and mitochondrial tryparedoxin peroxidase (mTXNPx) expression and a decrease of 50.0% in cytoplasmic tryparedoxin peroxidase (cTXNPx) in infected midguts (**Figure 3C**).

### The Midgut Cellular Response Begins in the Early Stages of *S. culicis* Infection

**Figure 4:**
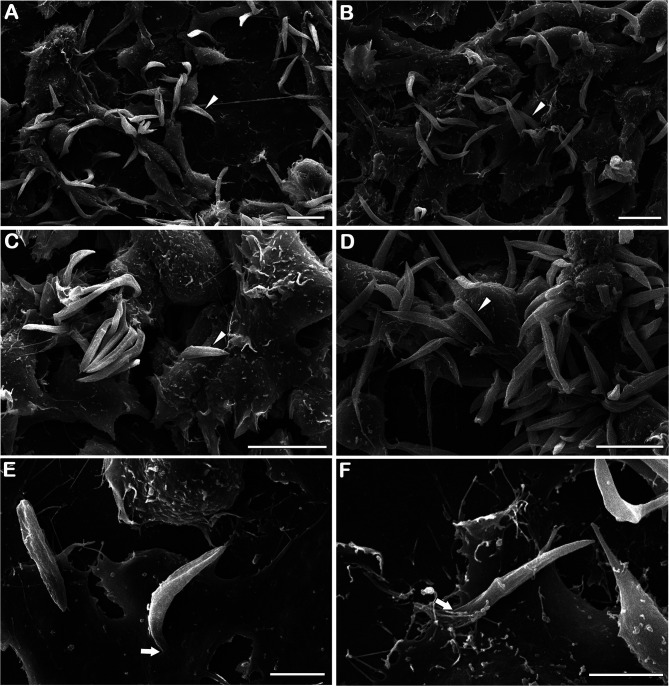
*Strigomonas culicis* infects Aag2 cell culture by flagellum insertion in epithelial cells. Scanning electron microscopy analysis of **(A, C, E)** wild type (WT) and **(B, D, F)** hydrogen peroxide (H_2_O_2_)-resistant (WTR) interaction with Aag2 at 24 h. Arrowheads point to adhered parasites, being possible to observe an increase in WTR-infected groups. Despite that, parasite–host interaction occurs similarly independent of the strain used mainly by flagellum insertion (arrows). Scale bars represent 10 µm **(A–D)** and 5 µm **(E, F)**. Representative micrographs of three independent experiments.

**Figure 5:**
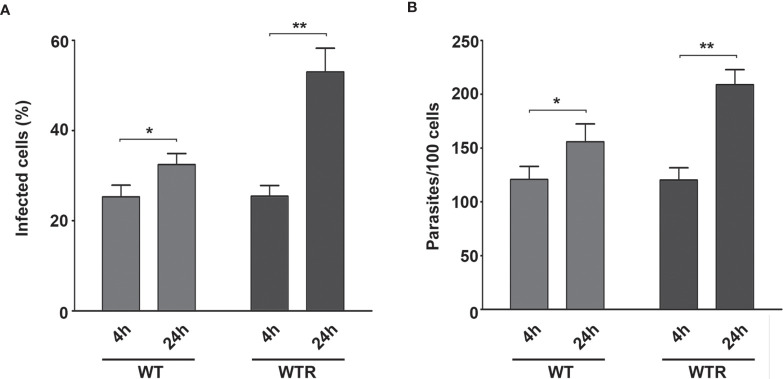
*Strigomonas culicis* hydrogen peroxide (H_2_O_2_)-resistant (WTR) had higher infection rates in Aag2 cell culture at 24 (h) Epithelial cells were infected with wild type (WT) and WTR strains for 4 (h) After that, non-adhered parasites were discarded, and Aag2 cell culture was maintained until 24 (h) **(A)** The percentage of infected cells (*p = 0.01, **p = 0.004) and **(B)** the number of adhered parasites per 100 cells (*p = 0.01, **p = 0.003) were calculated at both time points. Significant p-values were obtained by Mann–Whitney test, and error bars represent the mean ± SD of at least four independent experiments.

Using Aag2 cell line, an interesting model for insect immune studies (Barletta et al., 2012), we evaluated the early steps (4 and 24 h post-infection) of *S. culicis* infection in midgut cells. Scanning electron microscopy analysis revealed that parasite–host interaction occurs in a similar manner independently of the strain used mainly by flagellum insertion in epithelial cells and in some cases with protist body participation. On the other hand, there is a remarkable difference in the level of infection between the strains, especially at 24 h (**Figure 4** and **Figure S3**). Quantitative analysis revealed that both strains interact similarly in an early time, with up to 25% infected cells and an average of 120.7 parasites per 100 cells. Comparing 4 and 24 h, an increase in WT and WTR infections was detected. The percentage of WT-infected cells increased 1.2-fold in the later time point, while in the WTR group, this increase was more expressive, reaching up to 2.0-fold. The number of parasites per 100 cells was also higher, detecting an increase of 1.3- and 1.7-fold in WT and WTR infections, respectively (**Figure 5**).

**Figure 6:**
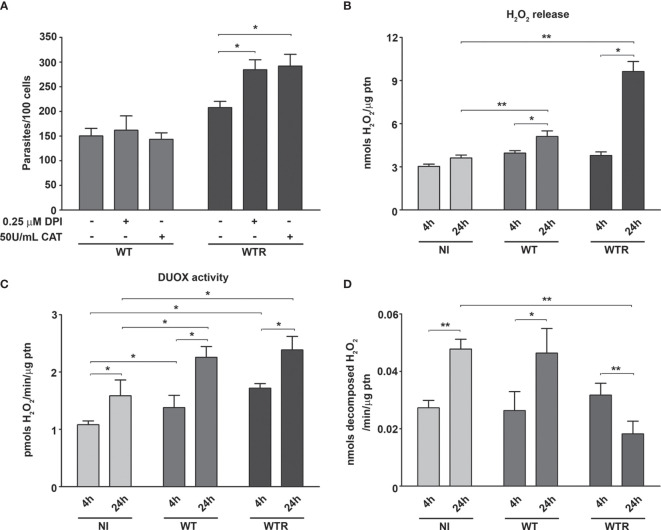
Host catalase inhibition was responsible for the high hydrogen peroxide (H_2_O_2_) production in *Strigomonas culicis* H_2_O_2_-resistant (WTR)-infected Aag2 cell cultures at 24 (h) **(A)** Number of adhered parasites per 100 cells at 24 h (*p = 0.01). Aag2 cells were treated with 0.25 µM diphenylene iodonium (DPI) or 50 U/ml catalase for 2 h before the infection. After that, the infection with wild-type (WT) and WTR strains was performed as described above. **(B)** Total H_2_O_2_ production measurement (*p = 0.05, **p = 0.03) and **(C)** ionomycin-derived dual oxidase (DUOX) activity in non-infected (NI) and WT- and WTR-infected cultures at 4 and 24 h (*p = 0.03). Enzyme activity was performed through the production of H_2_O_2_ in cell culture incubated with 1 µM ionomycin. Aag2 cells were mechanically dissociated, and the protein content was assessed after H_2_O_2_ production assay. **(D)** Catalase activity in cell culture homogenates (*p = 0.02, **p = 0.01). Significant p-values were obtained by Mann–Whitney test, and error bars represent the mean ± SD of at least three independent experiments.

After that, non-lethal doses (data not shown) of pro-oxidant and antioxidant molecules were tested in Aag2 cell cultures, aiming to evaluate their participation to *S. culicis* establishment. The treatment of Aag2 with 0.25 µM DPI or 50 U/ml catalase did not impact cell infection by both strains at 4 h (**Figures S4A, B**). At 24 h, both compounds increased the number of parasites per 100 cells up to 1.4-fold in the WTR-infected group, while no effect was detected in the WT-infected one (**Figure 6A** and **Figure S4C**). The evaluation of H_2_O_2_ release by Aag2 revealed that ROS production was time-dependent, increasing 1.3- and 2.5-fold in WT and WTR-infected cells, respectively (**Figure 6B**). Our data showed that both infections induced DUOX activity up to 1.6-fold at 4 h. DUOX activity was upregulated in all groups comparing both time points, being the most prominent increase found in infected groups (1.6- and 1.4-fold in WT and WTR, respectively). Despite the similar catalase activity of NI and infected groups at 4 h, an increase of 1.7-fold in NI and WT groups was observed after 24 h. In contrast, Aag2 cells had a significant decrease of 53.1% in this enzymatic activity comparing both time points of WTR infection (**Figures 6C, D**).

**Figure 7:**
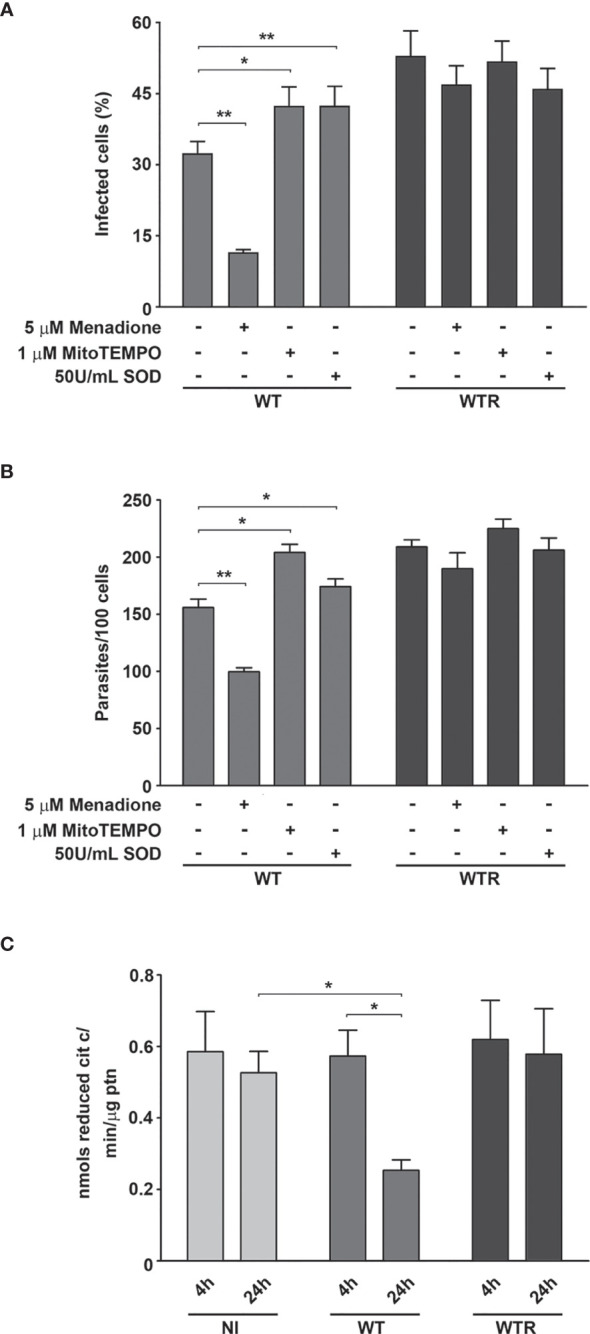
Wild type (WT) infection led to a host mitochondrial impairment. **(A)** Percentage of infected cells and **(B)** number of adhered parasites per 100 cells at 24 h (*p = 0.02, **p = 0.01). Aag2 cells were treated with 5 µM menadione, 1 µM mitoTEMPO, or 50 U/ml superoxide dismutase (SOD) for 2 h before the infection. After that, the infection with WT and hydrogen peroxide (H_2_O_2_)-resistant (WTR) strains was performed as described above. **(C)** Mitochondrial complex I–III activity of non-infected (NI) and WT- and WTR-infected cultures at 4 and 24 h (*p = 0.02). Significant p-values were obtained by Mann–Whitney test, and error bars represent the mean ± SD of at least three independent experiments.

To evaluate the contributions of Aag2 mitochondrial metabolism and superoxide radical production during parasite interaction, the treatment of epithelial cells with 5 µM menadione, 1 µM mitoTEMPO, and 50 U/ml SOD was performed. Menadione strongly impacts WT infection, decreasing 44.5% infected cells and 27.5% the number of adhered parasites at 4 h (**Figures S4D, E**). This phenotype was exacerbated after 24 h, and the decrease of infected cells and parasites per 100 cells reaches 63.1% and 36.0%, respectively. On the other hand, pretreatment with mitoTEMPO and SOD increased the WT infection parameters up to 1.4-fold at 24 h (**Figures 7A, B**). We also observed a significant impairment in Aag2 mitochondrial complex I–III activity caused by WT infection at 24 h. No differences were observed in infection quantification or in complex I–III activity of Aag2 cultures infected with WTR (**Figure 7C**).

### *S. culicis* Infection in *A. aegypti* Midgut Stimulates the Differential Expression of Antimicrobial Peptides Through the Immune Deficiency Pathway

**Figure 8:**
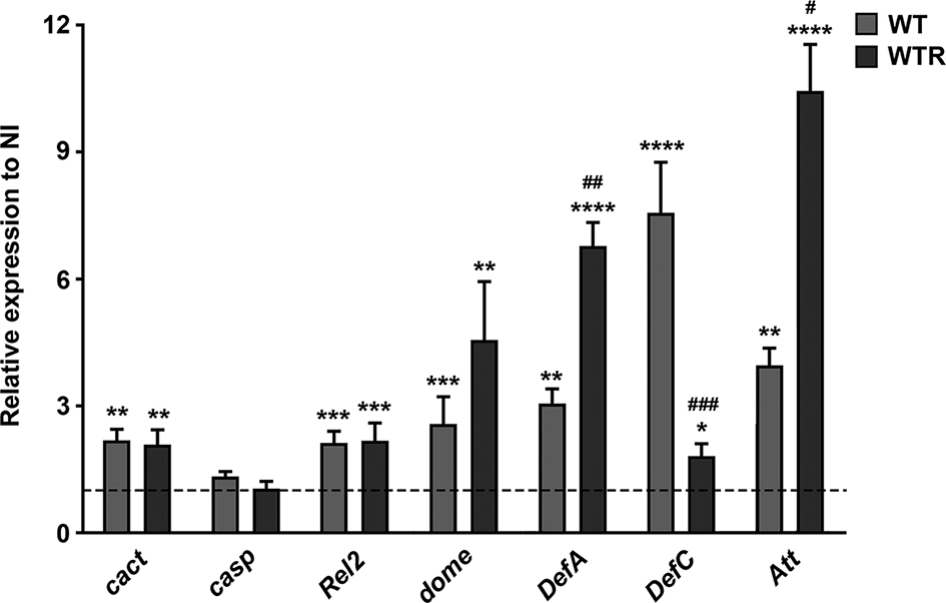
*Strigomonas culicis* strains stimulated innate immune response, mainly by the Immune deficiency (IMD) and Janus kinase/signal transducer and activator of transcription (JAK–STAT) signaling pathways, during midgut infection. The transcript levels were evaluated in non-infected (NI), wild-type (WT) and hydrogen peroxide (H_2_O_2_)-resistant (WTR)- infected midguts 4 dpi. Gene expression was normalized using the transcript levels of the NI group (dashed line). Ribosomal protein S7 was used as an endogenous control. Significant p-values were obtained by Mann–Whitney test comparing NI and *S. culicis*-infected groups (*p = 0.02, **p = 0.003, ***p = 0.002, ****p = 0.001) or WT- and WTR-infected groups (^#^p = 0.02, ^##^p = 0.01, ^###^p = 0.002), and error bars represent the mean ± SEM of three independent experiments.

To assess whether *S. culicis* strains trigger an innate immunological response during midgut infection, we determined gene expression of key molecules in the immune pathways Toll, Immune deficiency (IMD), and Janus kinase/signal transducer and activator of transcription (JAK/STAT). Our results demonstrate that both strains modulate the expression of all immune pathways analyzed. The gene expression of the negative regulator of Toll pathway *cact* was upregulated, but the expression of the negative regulator of IMD pathway *casp* was not altered, suggesting an inactivation of Toll pathway after *S. culicis* infection. In contrast, the transcription factor *Rel2* was similarly increased by WT and WTR strains, reaching 2.2-fold in relation to NI; in addition, the receptor of JAK–STAT pathway *domeless* (*dome*) was also upregulated by both strains, suggesting activation of both immune pathways. Except for *DefC*, the transcriptional increase of antimicrobial peptide (AMP) genes was greater in WTR-infected midguts. WT-infected midguts showed an increase of 3.0- and 7.5-fold in the expression of *DefA* and *DefC*, compared to uninfected control, while WTR-infected tissues presented an increase of 6.8- and 1.8-fold in each of those AMPs, respectively. Additionally, the gene expression of *Att* increased 3.9- and 10.4-fold in WT- and WTR-infected midguts, respectively (**Figure 8**).

### *S. culicis* Infection Impairs *A. aegypti* Reproductive Fitness

**Figure 9:**
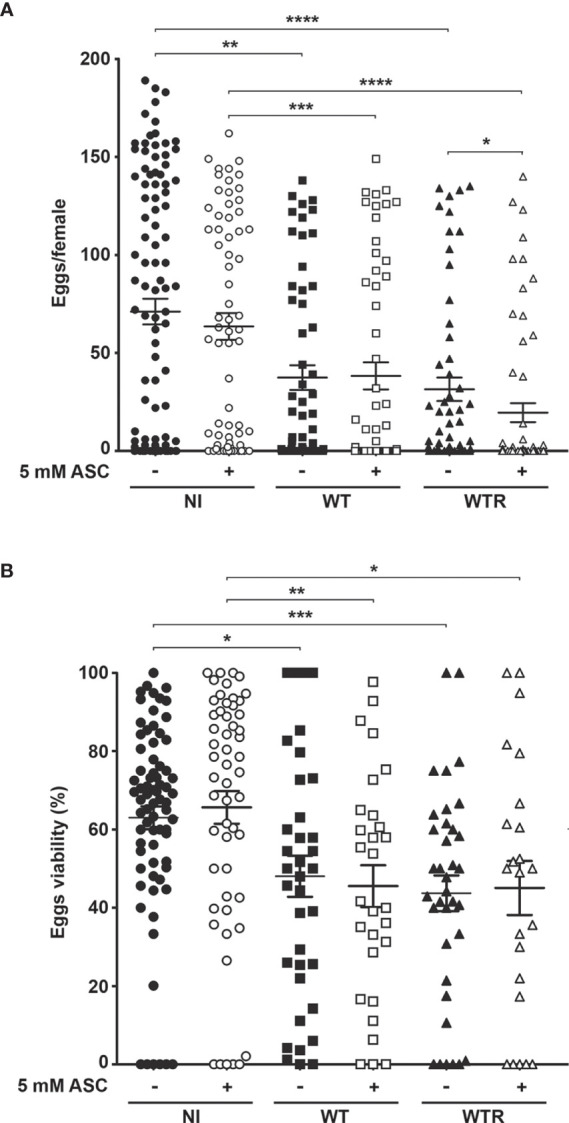
*Strigomonas culicis* infection disrupts the reproductive fitness of *Aedes aegypti* females. **(A)** Egg production (*p = 0.006, **p = 0.003, ***p = 0.002, ****p = 0.001) and **(B)** viability (*p = 0.01, **p = 0.002, ***p = 0.001) were evaluated in non-infected (NI), wild-type (WT) and hydrogen peroxide (H_2_O_2_)-resistant (WTR)- infected females fed with 10% sucrose or 10% sucrose + 5 mM sodium L-ascorbate (ASC) *ad libitum*. Synchronized oviposition was stimulated 4 dpi. Significant p-values were obtained by Mann–Whitney test, and error bars represent the mean ± SEM of two independent experiments.

To determine whether *S. culicis* infection and the exacerbated ROS production affect *A. aegypti* reproductive fitness, the first gonotrophic cycle was analyzed. The overall results showed that both strains cause similar losses in egg production and viability after sucrose and ASC feeding (**Figure 9**). WT and WTR led to a reduction in egg production up to 55.7% in sugar-fed and 61.8% in ASC-fed groups compared to diet-matched NI mosquitoes. The addition of antioxidant potentiated the harmful effects caused by WTR infection, decreasing egg production 37.9% in relation to infected females (**Figure 9A**). NI females presented more than 60.0% egg viability in both diet groups, while midgut infection decreased this value until 30% (**Figure 9B**). To confirm that reproductive disruption was derived from *S. culicis* infection, we evaluated this process in Apo-infected females. The presence of Apo strain did not impact egg production in both diet strategies; however, a reduction of 20.2% in egg viability was observed after ASC feeding (**Figure S5**).

*S. culicis* infection, especially by WTR, had a strong impact on oviposition efficiency, decreasing the median of eggs laid per female more than 10-fold according to the diet used. Efficiency was determined according to the median of eggs laid by the diet-matched uninfected group. The proportion of efficient females was lowered during midgut infection, especially by WTR, which reduced the percentage from 50.0% in NI to 19.3% and 15.9% in sugar- and ASC-fed, respectively. Regarding null females, we observed a similar increase comparing NI and infected groups fed with sugar; however, when the ASC-enriched diet was provided, there is a greater increment in this percentage, reaching 63.5% in WTR infection. Curiously, the percentage of infertile females was affected only in WTR groups, increasing from 9.3% in NI to 21.7% in ASC-fed females (**Table 1**).

**Table 1:** Oviposition efficiency of *A. aegypti* females infected with *S. culicis* strains in the presence or not of ASC-enriched diet.

	N^a^	Median egg production	% of efficient females^b^	% of null females^c^	% of infertile females^d^
**10% sucrose diet**					
**NI**	101	69.0	50.0	27.7	8.2
**WT**	57	7.0	28.1	35.1	7.4
**WTR**	57	5.0	19.3	36.8	13.9
**10% sucrose + 5 mM ASC diet**					
**NI**	66	61.5	50.0	18.2	9.3
**WT**	56	1.5	30.4	48.2	10.3
**WTR**	63	0.0	15.9	63.5	21.7

a N, number of females.

b Females that produced a number of eggs equal to or greater than the median production of control mosquitoes were considered efficient. Efficiency was measured according to median of diet-matched uninfected group.

c Females that did not lay any eggs were considered null.

d Females with unhatched eggs.

A. aegypti, Aedes aegypti; ASC, sodium L-ascorbate; NI, non-infected; S. culicis, Strigomonas culicis; WT, wild-type; WTR, hydrogen peroxide (H_2_O_2_)-resistant (WTR).

**Figure 10:**
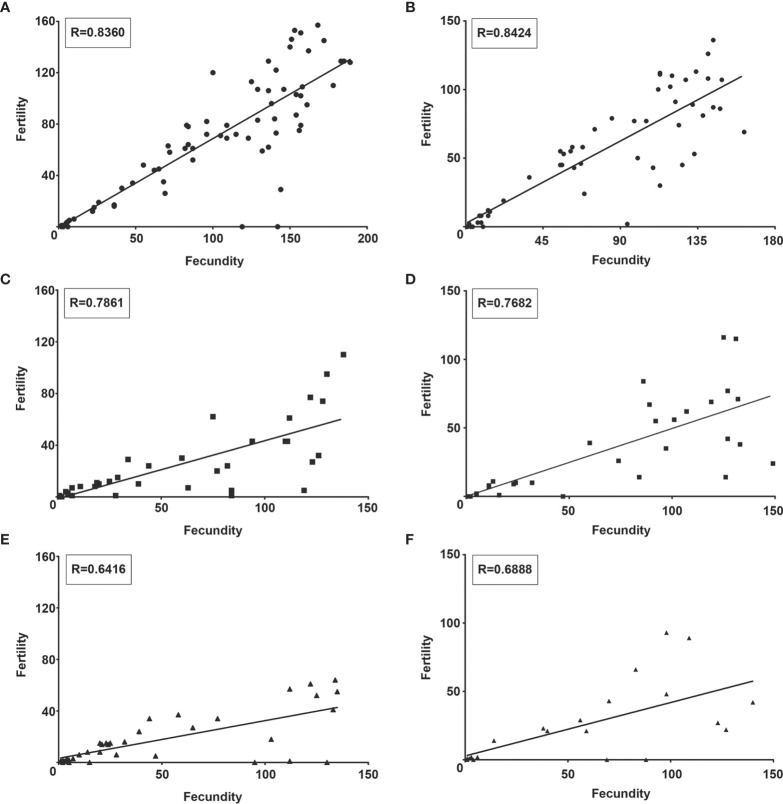
*Strigomonas culicis* infection impaired the correlation between fecundity and fertility in *Aedes aegypti* females. Correlation analysis of **(A, B)** non-infected and **(C, D)** wild type (WT)- and **(E, F)** hydrogen peroxide (H_2_O_2_)-resistant (WTR)- infected females fed with **(A, C, E)** 10% sucrose or **(B, D, F)** 10% sucrose + 5 mM sodium L-ascorbate (ASC) *ad libitum*. Analysis was made by Correlation of Spearman using data of two independent experiments. R value of each correlation is described in the boxes.

Although *S. culicis*-infected females maintained the positive correlation between fecundity (numbers of eggs laid) and fertility (number of eggs hatched), the strength of this correlation, as found in NI mosquitoes, was lost after the infection. As observed in **Figure 10A**, the control group had a homogeneous distribution and linear relationship (R = 0.8360, p < 0.0001). In contrast, infected mosquitoes had a dispersed distribution and linearity impairment after WT (R = 0.7861, p < 0.0001) and WTR (R = 0.6416, p < 0.0001) infections (**Figures 10C, E**). Moreover, antioxidant feeding did not impact fecundity and fertility correlation when compared to sugar-fed females (**Figures 10B, D, F**).

## Discussion

The intestinal epithelium of insects is in constant contact with microorganisms, which can be beneficial or harmful to the host. Gut health and functions are maintained through several mechanisms that can efficiently remove foreign pathogenic microorganisms, preserving the resident microbiota (Bai et al., 2021). ROS has a role in insect innate immune response as a potent pathogen-killing agent. In *A. aegypti*, bacterial proliferation is stimulated by the downregulation of ROS levels, culminating in increased insect mortality upon infection (Oliveira et al., 2011). A similar phenomenon is observed after dipteran infection with protists. *P. berghei* development in mosquitoes is controlled by the reduction of H_2_O_2_ detoxification pathways in the midgut (Molina-Cruz et al., 2008), while *Trypanosoma brucei* infection elicits an increased gene expression of inducible nitric oxide synthase (iNOS) and DUOX in parasite-resistant flies (Weiss et al., 2013). In previous studies of our group, we demonstrated that the use of *S. culicis* H_2_O_2_-resistant parasites and/or diet supplementation with antioxidant enhanced midgut infection for up to 11 dpi. The WTR strain is more resistant than the WT to pro-oxidant molecules, such as H_2_O_2_, menadione, and antimycin A, pointing to a cross-resistance between different reactive species. In addition, this strain presented a significantly lowered H_2_O_2_ release and increased abundance and activity of antioxidant enzymes, highlighting a more efficient response to the oxidative challenge (Bombaça et al., 2017; Bombaça et al., 2020). Nonetheless, characterization of a ROS-mediated immunological response was not assessed in *S. culicis*-infected mosquitoes.

Here, *A. aegypti* mosquitoes were orally infected with *S. culicis* strains, and ROS production was evaluated at 4 dpi. At this time point, an initial decrease in the number of parasites per midgut was described, probably reflecting the immune mechanisms elicited in the insect (Corrêa-Da-Silva et al., 2006; Bombaça et al., 2017). Interestingly, WTR strain specifically triggers a DUOX-dependent ROS response, while the WT strain induces the production of superoxide radical in detriment to other oxidizing species. Altogether, these results suggest the existence of a strain-specific response in the midgut epithelial cells. Our approach *in vitro* using Aag2 cells confirmed our findings in mosquito midgut, regulating WT and WTR infections through the host’s cell treatment with DPI, menadione, and specific antioxidants. Since the main ROS production pathway does not participate in the immunological response against WT infection, other cellular processes that lead to superoxide radical production were investigated. Mitochondrion is the organelle involved not only in aerobic energy transduction to allow ATP synthesis but also in redox balance, representing one of the major sources of ROS (Kowaltowski et al., 2009). Reductions in mitochondrial membrane potential and ROS generation were able to decrease *A. gambiae* resistance to *P. berghei* infection, increasing the number of oocysts per midgut (Gonçalves et al., 2012). Similarly, WT infection impairs CS and complex I–III activities, which denote a commitment to oxidative phosphorylation. In parallel, increased infection rates were found after the Aag2 treatment with mitoTEMPO, a specific mitochondrial ROS scavenger. Altogether, these results suggest that WT strain induced an immune response sustained by mitochondrial superoxide production increase.

As previously mentioned, ROS are pathogen-killing molecules, and their uncontrolled production destroys the microbiota homeostasis, leading to disease development in the host (Bai et al., 2021). The reduced expression of immune-regulated catalase causes high mortality rates in flies after oral infection, highlighting the importance of these reactive species for the host survival during continuous gastrointestinal exposure (Ha et al., 2005). Our analysis of Aag2 infection showed a time-dependent increase of DUOX activity in all evaluated groups, reflecting only in H_2_O_2_ generation by WTR-infected cells. In the NI and WT groups, an increase in catalase activity was detected comparing 4 and 24 h, indicating a ROS-protective mechanism to prevent their overproduction. Preparing the mosquitoes for elevated ROS release and avoiding the host damage, catalase was upregulated in midguts of *A. aegypti* and *A. gambiae* after blood meal, peaking 24 h after feeding and returning to basal levels after complete digestion (Magalhaes et al., 2008; Molina-Cruz et al., 2008; Oliveira et al., 2017).

Here, a downregulation of catalase activity was observed in infected midguts and Aag2 cultures infected with the WTR strain, reinforcing the elevated H_2_O_2_ detection. *P. berghei*-infected midguts showed no catalase expression and lower enzyme activity in a specific response to tissue invasion by ookinetes, culminating in higher local levels of H_2_O_2_ (Molina-Cruz et al., 2008). In parallel, catalase silencing in the midgut of *Anopheles aquasalis* leads to increased *Plasmodium vivax* infection, showing that the model of ROS-induced parasite killing is species-dependent. This work also proposes a modulation of the host detoxifying pathways by the parasite probably as a mechanism to decrease competitive microbiota and inhibit the immune system to improve its own development (Bahia et al., 2013). Catalase-encoding gene is classically known to be absent in Trypanosomatidae species, except by some monoxenous genera of Leishmaniinae clade (Kraeva et al., 2017). To handle this question, there are several additional enzymes in these protists responsible for H_2_O_2_ detoxification, such as tryparedoxin peroxidase and trypanothione-dependent enzymes. Here, we showed that mTXNPx and TR mRNA levels were more increased in WTR than in WT during midgut infection. The positive regulation of both enzymes in *S. culicis* was previously described, as a consequence of H_2_O_2_ exposure during resistance selection (Bombaça et al., 2017; Bombaça et al., 2020). The overexpression of detoxifying genes, especially in dixenous trypanosomatids, can act as a preparatory mechanism to deal with oxidative burst, resulting in increased host infection (Atwood et al., 2005; Piacenza et al., 2013). Although *S. culicis* does not have in its genome any catalase-encoding gene, the expression of an ectopic protein could also contribute to the induction of H_2_O_2_ resistance. A previous report showed that monoxenous *Crithidia fasciculata*, a catalase-carrying trypanosomatid, proliferates in the tsetse fly upon injection into the hemocoel, while catalase-lacking protists failed to survive in the same environment (Ibrahim and Molyneux, 1987). These findings suggest that catalase expression may be beneficial to monoxenous trypanosomatids, since ROS production is an important mechanism of insects’ innate immune defense. Although the ectopic expression of catalase in *T. brucei* and *T. cruzi* increases the cell resistance to H_2_O_2_ stress and the invertebrate infection, it failed to induce a significant increase of parasite virulence to vertebrate host. Similarly, catalase-expressing *Leishmania mexicana* had their development in insects and mice severely compromised. Taken together, these data support the hypothesis that the absence of catalase in some trypanosomatids is essential for the emergence of a dixenous life cycle due to the regulatory role of H_2_O_2_ in the transition from insect to the mammalian stages (Freire et al., 2017; Kraeva et al., 2017; Horáková et al., 2020; Sádlová et al., 2021). Curiously, tsetse flies have not been found hosting catalase-carrying monoxenous trypanosomatids in the wild, although species of Leishmaniinae clade are widely identified in dipteran insects. In this case, the presence of catalase could facilitate the proliferation of these trypanosomatids in tsetse flies (as shown by the above data), leading to higher infection of the insects and, eventually, to their death (Ibrahim and Molyneux, 1987; Týč et al., 2013; Kraeva et al., 2017; Horáková et al., 2020).

Our overall results indicate that WTR triggers a widely DUOX-mediated response, in addition to an oxidative burst enlargement due to catalase activity inhibition. Unexpectedly, our previous work showed a discrete difference in mortality of females after midgut infection and showed that *A. aegypti* controls the WTR population even in ASC-fed conditions (Bombaça et al., 2017). We raised the question whether infection management, mainly when caused by WTR, may come with a cost, such as an adverse effect on reproductive fitness. Data indicate that both strains cause similar impairment to females’ fecundity and fertility, except by oviposition efficiency and female infertility that were most damaged in WTR-infected. Also, we observed a more expressive loss in the correlation between fecundity and fertility probably as a consequence of an increased number of unhatched eggs. Corroborating this, in the correlation analysis performed without infertile females, the R value was similar to that of NI and WT groups (data not shown). Some authors point to the oxidative balance as a crucial factor to the reproductive capacity of mosquitoes. *A. gambiae* strains with variable H_2_O_2_ levels and refractory/susceptible infection profiles showed a decline in fecundity and fertility due to cumulative oxidative damage. Same results were found following the oral administration of paraquat, a pro-oxidant molecule, in mosquitoes. These studies attested to the importance of an antioxidant system, through manipulation of catalase activity or NADPH pool, in redox state homeostasis and consequent fecundity commitment (Kumar et al., 2003; DeJong et al., 2007; Champion and Xu, 2018). It is important to mention that although an antioxidant-enriched diet has been offered to *S. culicis*-infected females, the protection against fecundity disruption was not achieved. Instead, ASC feeding potentiated the number of null females especially after WTR infection. Further analyses should be performed to determine whether the oxidative stress triggered by *S. culicis* midgut infection is also present in other host tissues, such as fat body, hemocoel, and ovaries. Despite that, the direct correlation between the increased reproductive cost and the elevated parasite load cannot be discarded, suggesting a ROS-independent mechanism in ASC-fed mosquitoes.

Regarding the three major signaling pathways responsible for controlling mosquito innate immune response (De Gregorio et al., 2002; Dostert et al., 2005), *S. culicis* infection positively modulated IMD and JAK–STAT. Previous work showed that *casp* gene silencing resulted in an IMD pathway-mediated immune defense, producing a *Plasmodium falciparum*-refractory profile in mosquitoes (Garver et al., 2009; Garver et al., 2012). The IMD pathway also participates in invertebrate response against trypanosomatids. RNA interference targeting *casp* positively impacts *Leishmania* spp. numbers in the gut of sand flies. Similarly, the mutagenesis of *Rel2* by CRISPR/Cas9 generates a more permissive phenotype in insect vector, presenting higher loads of promastigotes (Telleria et al., 2012; Louradour et al., 2019). Hamilton et al. (2015) described that *Jaenimonas drosophilae*, a trypanosomatid found naturally infecting *Drosophila fallen*, is able to establish infections in larvae of different species of *Drosophila*. In this case, the infection persists throughout the life of the fly and even through metamorphosis, when the insect’s gut undergoes intense rearrangements that can compromise the infection. *J. drosophilae* infection culminates in impaired host’s fecundity and innate immune system activation, disrupting the idea that monoxenous organisms are benign to their insect hosts. In contrast to our results and with literature (Hu and Aksoy, 2006; Telleria et al., 2012; Louradour et al., 2019), the authors demonstrated that the infection in IMD-deficient *D. melanogaster* did not increase *J. drosophilae* load, which suggests that the immune activation observed could be an indirect by-product of other gut microorganisms affected by/or interacting with the protist.

AMPs are positively charged peptides that contribute to the innate immunological system, being controlled by Toll and IMD signaling pathways, participating in the response against bacteria, yeast, fungi, and *Plasmodium* (Hanson and Lemaitre, 2020). Our data pointed to an intense overexpression of AMPs in *S. culicis*-infected mosquitoes 4 dpi. The gene expression of *cact* (a suppressor of Toll pathway) was increased, suggesting a negative regulation of Toll. At the same time, the transcription factor *Rel2* was upregulated, which indicates that the IMD pathway is the main one responsible for AMP-dependent response against *S. culicis* strains. In *D. melanogaster*, the chronic activation of the IMD pathway leads to overwhelming expression of AMPs, generating an imbalance in intestinal microbiota homeostasis and growth of opportunistic pathogens (Ryu et al., 2008). WTR infection stimulates an elevated expression of *DefA* and *Att*, which can favor foreign bacterial development and uracil secretion. The role of JAK–STAT pathway during parasite infection was also demonstrated by some authors. Silencing of the transcription factor STAT-coding gene enhances the overall infection of *P. berghei* in mosquito midgut, increasing oocyst survival. In contrast, silencing of the STAT suppressor reduced parasite infection through high NOS levels (Gupta et al., 2009). In a similar manner, knockdown of STAT increased the infection in the midgut of *A. aquasalis* by early stages of *P. vivax* (Bahia et al., 2011). Because high levels of *dome* transcripts were detected in both *S. culicis* strains, we believe that this pathway is also functional, contributing to oxidative burst.

The antiviral activity of AMPs was demonstrated in *A. aegypti* infected with *Wolbachia*, in which the bacteria lead to the overproduction of ROS, defensins, and cecropins, conferring resistance to Dengue virus. Depletion of defensin C and cecropin D compromised the *Wolbachia*-induced resistance to viral infection, while the ectopic expression of defensin A and cecropin A inhibits viral proliferation (Pan et al., 2012). In *A. aegypti*, the JAK–STAT pathway also triggers an antiviral response. The suppression of *dome* increased the Dengue virus detection, while the mosquitoes became more resistant to viral infection through the exacerbation of this pathway, decreasing viral load and dissemination (Souza-Neto et al., 2009; Jupatanakul et al., 2017). Interestingly, gene expression of several antioxidant enzymes is upregulated during Dengue infection, controlling the immunological ROS response to allow viral development. Thus, mosquitoes with reduced oxidative burst had an expansion of viral load, while the enlargement of ROS production, *via* catalase silencing, decreases the infection prevalence (Liu et al., 2016; Oliveira et al., 2017; Cheng et al., 2021). Altogether, these findings suggest that innate immune manipulation and oxidative stress exacerbation are important strategies to control viral development in *A. aegypti*. Here, we demonstrated that WTR naturally exacerbates ROS production in the midgut, promoting an increase in innate immune response of the insect, an interesting checkpoint for the mosquito–virus interaction. Given that *S. culicis* is classically known as a non-pathogenic trypanosomatid for humans that usually colonizes *A. aegypti*, the use of the H_2_O_2_-resistant strain could be a promising strategy for the biological control of virus infection.

## Data Availability Statement

The original contributions presented in the study are included in the article/**Supplementary Material**. Further inquiries can be directed to the corresponding authors.

## Author Contributions

ASB, AG, VB-R, FD, and RM-B designed and performed the experiments to determine ROS production in *A. aegypti* and Aag2 cell cultures. ASB, VE-V, MS, AB, and RM-B designed and participated in immune system evaluation. ASB, AG, LF, RB, and RM-B designed and performed fecundity and fertility assays. ASB, AG, and RM-B drafted the manuscript. All authors contributed to the article and approved the submitted version.

## Funding

Funding was provided by Coordenação de Aperfeiçoamento de Pessoal de Nível Superior (CAPES, Financial Code 001), Fundação de Amparo à Pesquisa do Rio de Janeiro (FAPERJ), Conselho Nacional de Desenvolvimento Científico e Tecnológico (CNPq), and Fundação Oswaldo Cruz (FIOCRUZ).

## Conflict of Interest

The authors declare that the research was conducted in the absence of any commercial or financial relationships that could be construed as a potential conflict of interest.

## Publisher’s Note

All claims expressed in this article are solely those of the authors and do not necessarily represent those of their affiliated organizations, or those of the publisher, the editors and the reviewers. Any product that may be evaluated in this article, or claim that may be made by its manufacturer, is not guaranteed or endorsed by the publisher.

## Supplementary Material

Supplementary Figure 1 Apo infection persistence in ASC-fed females increases ROS-dependent response. Analysis was performed in midguts of non-infected (NI) and Apo-infected females fed with 10% sucrose or 10% sucrose + 5 mM ASC *ad libitum* at 1 and 4 dpi (*P = 0.01, **P = 0.005, ***P = 0.01). Quantitative analysis of fluorescence microscopy was performed individually in 10-15 midguts per group. Significant p-values were obtained by Mann-Whitney test and error bars represent mean ± SEM of three independent experiments.

Supplementary Figure 2 DPI feeding decreased DHE staining in *S. culicis*-infected midguts. Analysis was performed in midguts of non-infected (NI), WT- and WTR-infected females fed with 10% sucrose or 10% sucrose + 10 µM DPI *ad libitum* 4 dpi. **(A)** DUOX activity of NI in response to incubation with 1 µM ionomycin, 5 µM DPI or 5 mM EGTA (*P = 0.05). Enzyme activity was performed through the production of H_2_O_2_ in pools of 20 guts. **(B)** Representative confocal images of midguts dissected and incubated with DHE. Scale bar represents 20 µm. Arrowheads indicate high staining in WTR-infected midgut. Representative images of three independent experiments. Significant p-values were obtained by Mann-Whitney test and error bars represent mean ± SEM of at least three independent experiments.

Supplementary Figure 3 *S. culicis* infects Aag2 cell culture by flagellum insertion in epithelial cells. Scanning electron microscopy analysis of **(A, C, D)** WT and (B,D,F) WTR interaction with Aag2 at 4 h. Arrowheads point adhered parasites, being possible to observe similar infection rates between WT and WTR infections. Parasite-host interaction also occurs similarly in both infections, mainly by flagellum insertion (arrows). Scale bars represent 10 µm **(A–D)** and 5 µm **(E, F)**. Representative micrographs of three independent experiments.

Supplementary Figure 4 Menadione treatment impairs WT infection in Aag2 cell cultures after 4 h. Epithelial cells were treated with 0.25 µM DPI, 50 U/mL catalase, 5 µM menadione, 1 µM mitoTEMPO or 50 U/mL SOD for 2 h before the infection. After that, the infection with WT and WTR strains was performed for 4 h. Non-adhered parasites were discarded and Aag2 cell culture fixed to infection evaluation or maintained until 24 h. Percentage of infected cells at **(A, D)** 4 or **(C)** 24 h (*P = 0.01). **(B, E)** Number of adhered parasites per 100 cells. Significant p-values were obtained by Mann-Whitney test and error bars represent mean ± SD of at least three independent experiments.

Supplementary Figure 5 Apo infection does not impair the reproductive fitness of *A. aegypti* females. **(A)** Egg production and **(B)** viability were evaluated in non-infected (NI) and Apo-infected females fed with 10% sucrose or 10% sucrose + 5 mM ASC *ad libitum* (*P = 0.01). Synchronized oviposition was stimulated 4 dpi. Significant p-values were obtained by the Mann-Whitney test (***P < 0.01) and error bars represent mean ± SEM of two independent experiments.

Supplementary Table 1 Oligonucleotides sequences used in qPCR assays.
