# Supplementary figures and images for: Aedes aegypti Infection With Trypanosomatid Strigomonas culicis Alters Midgut Redox Metabolism and Reduces Mosquito Reproductive Fitness
Source: Front Cell Infect Microbiol. 2021 Aug 13;11:732925. doi: 10.3389/fcimb.2021.732925 (PMC8414984; doi:10.3389/fcimb.2021.732925)

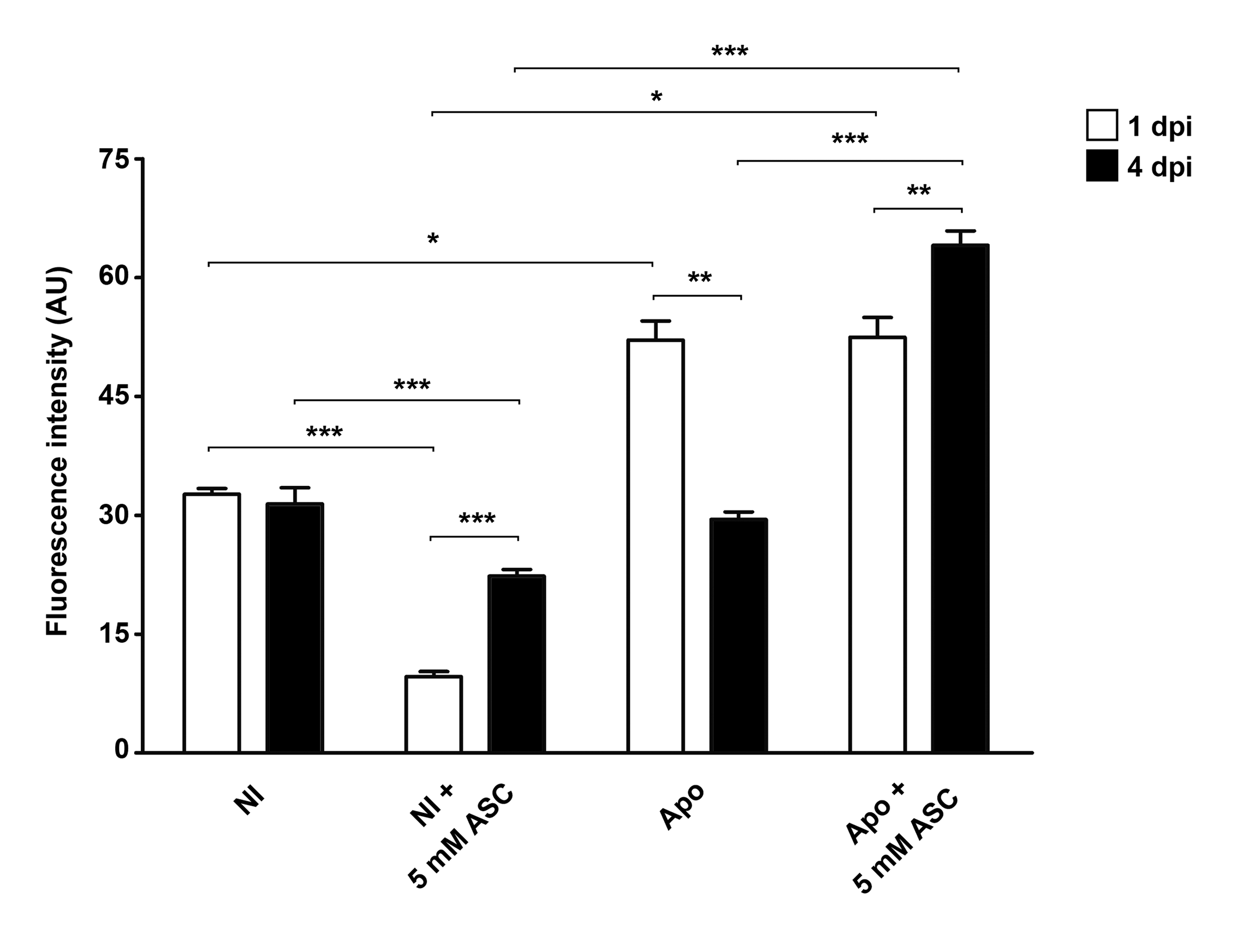

Supplement: Supplementary Figure 1 — Apo infection persistence in ASC-fed females increases ROS-dependent response. Analysis was performed in midguts of non-infected (NI) and Apo-infected females fed with 10% sucrose or 10% sucrose + 5 mM ASC ad libitum at 1 and 4 dpi (*P = 0.01, **P = 0.005, ***P = 0.01). Quantitative analysis of fluorescence microscopy was performed individually in 10-15 midguts per group. Significant p-values were obtained by Mann-Whitney test and error bars represent mean ± SEM of three independent experiments. [file Image_1.tif]

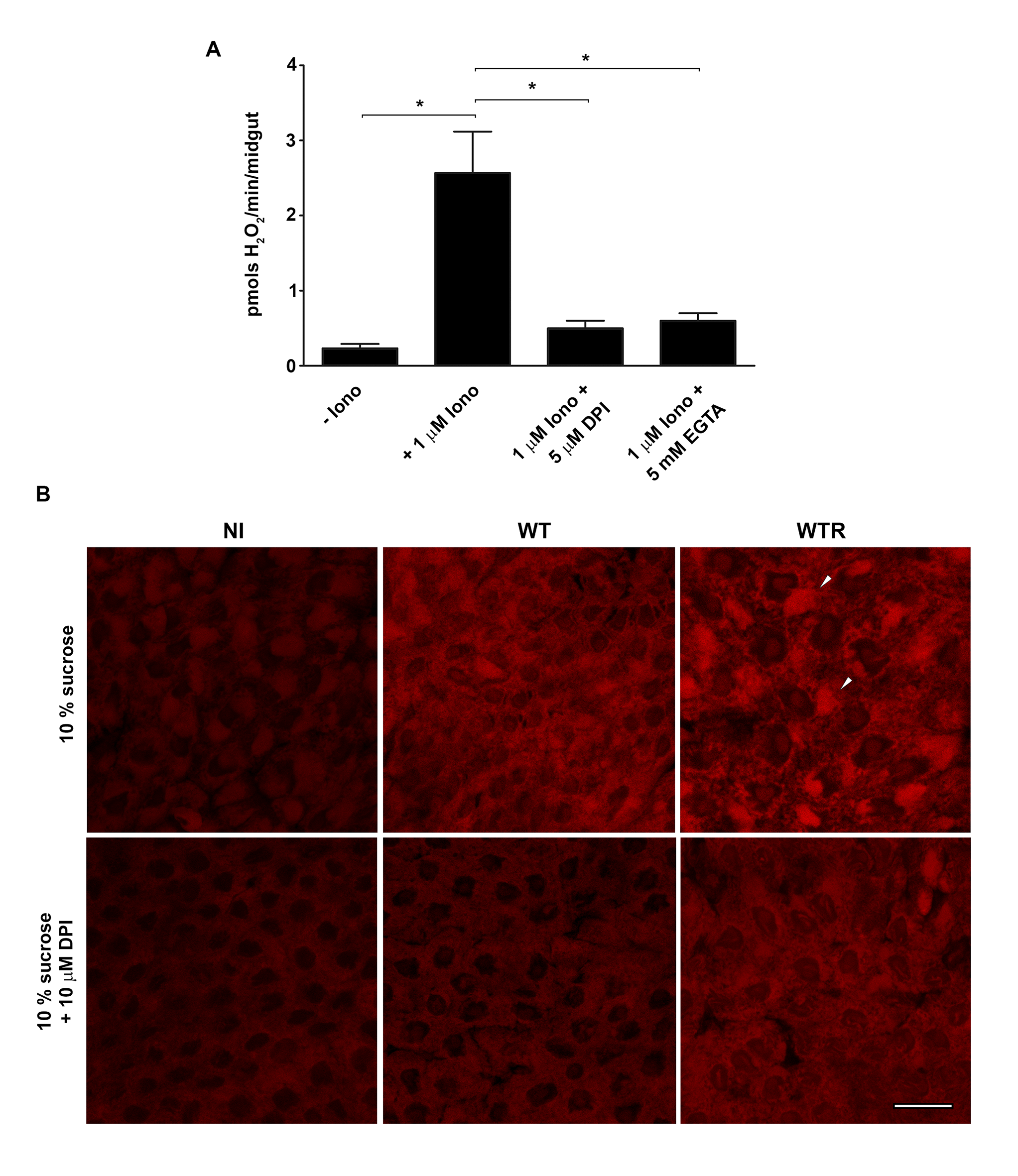

Supplement: Supplementary Figure 2 — DPI feeding decreased DHE staining in S. culicis-infected midguts. Analysis was performed in midguts of non-infected (NI), WT- and WTR-infected females fed with 10% sucrose or 10% sucrose + 10 µM DPI ad libitum 4 dpi. (A) DUOX activity of NI in response to incubation with 1 µM ionomycin, 5 µM DPI or 5 mM EGTA (*P = 0.05). Enzyme activity was performed through the production of H2O2 in pools of 20 guts. (B) Representative confocal images of midguts dissected and incubated with DHE. Scale bar represents 20 µm. Arrowheads indicate high staining in WTR-infected midgut. Representative images of three independent experiments. Significant p-values were obtained by Mann-Whitney test and error bars represent mean ± SEM of at least three independent experiments. [file Image_2.tif]

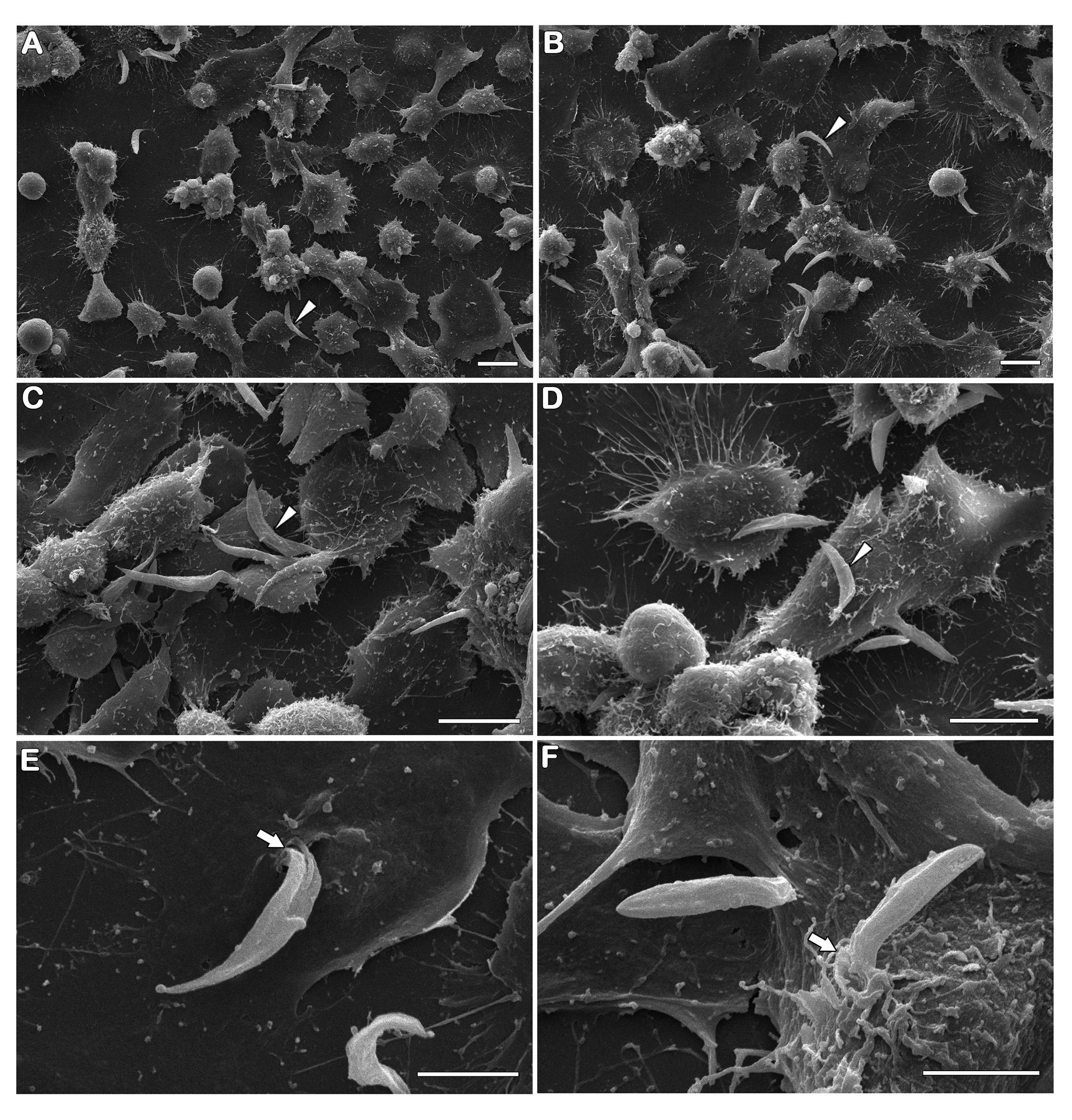

Supplement: Supplementary Figure 3 — S. culicis infects Aag2 cell culture by flagellum insertion in epithelial cells. Scanning electron microscopy analysis of (A, C, D) WT and (B,D,F) WTR interaction with Aag2 at 4 h. Arrowheads point adhered parasites, being possible to observe similar infection rates between WT and WTR infections. Parasite-host interaction also occurs similarly in both infections, mainly by flagellum insertion (arrows). Scale bars represent 10 µm (A–D) and 5 µm (E, F). Representative micrographs of three independent experiments. [file Image_3.tif]

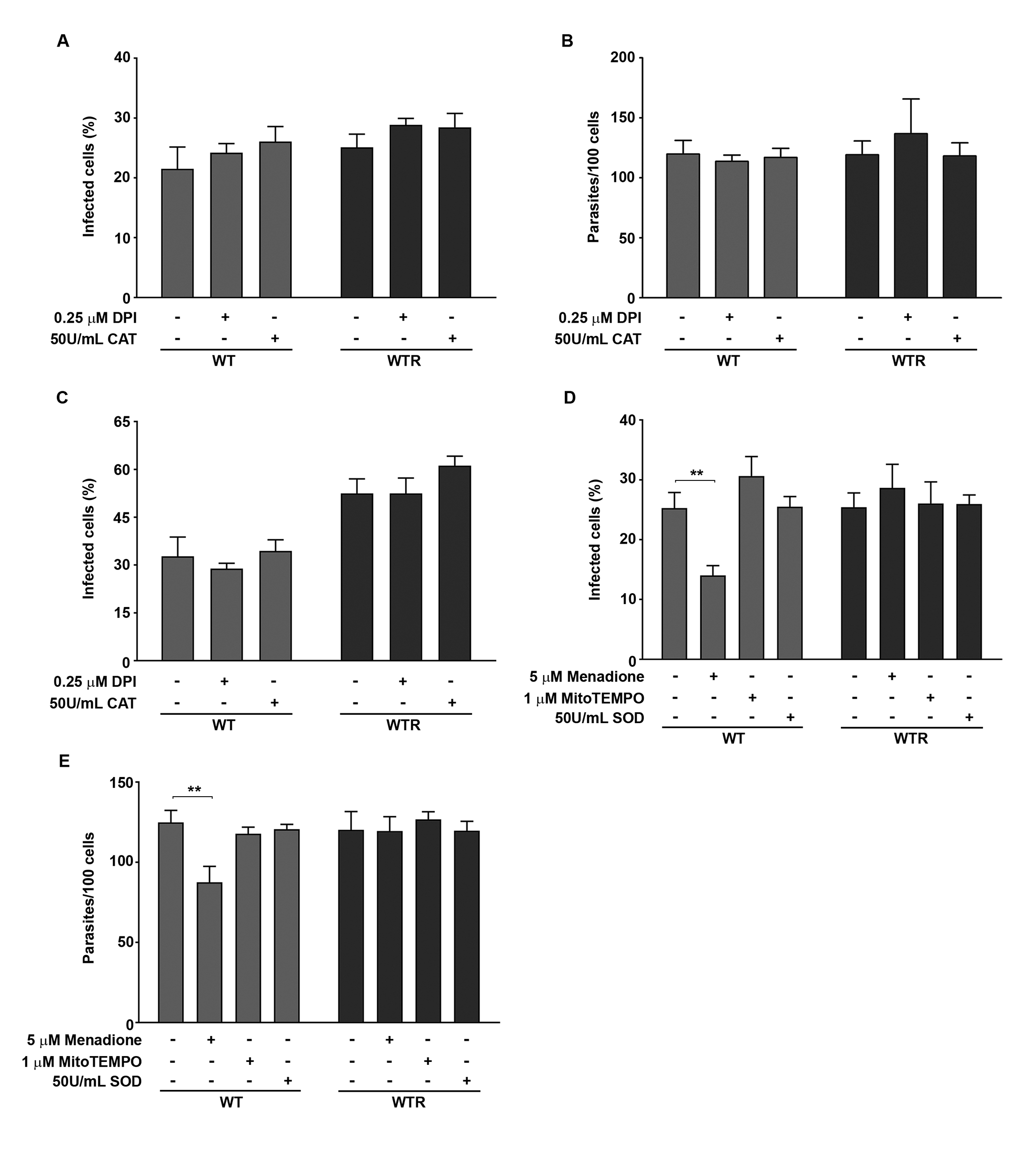

Supplement: Supplementary Figure 4 — Menadione treatment impairs WT infection in Aag2 cell cultures after 4 h. Epithelial cells were treated with 0.25 µM DPI, 50 U/mL catalase, 5 µM menadione, 1 µM mitoTEMPO or 50 U/mL SOD for 2 h before the infection. After that, the infection with WT and WTR strains was performed for 4 h. Non-adhered parasites were discarded and Aag2 cell culture fixed to infection evaluation or maintained until 24 h. Percentage of infected cells at (A, D) 4 or (C) 24 h (*P = 0.01). (B, E) Number of adhered parasites per 100 cells. Significant p-values were obtained by Mann-Whitney test and error bars represent mean ± SD of at least three independent experiments. [file Image_4.tif]

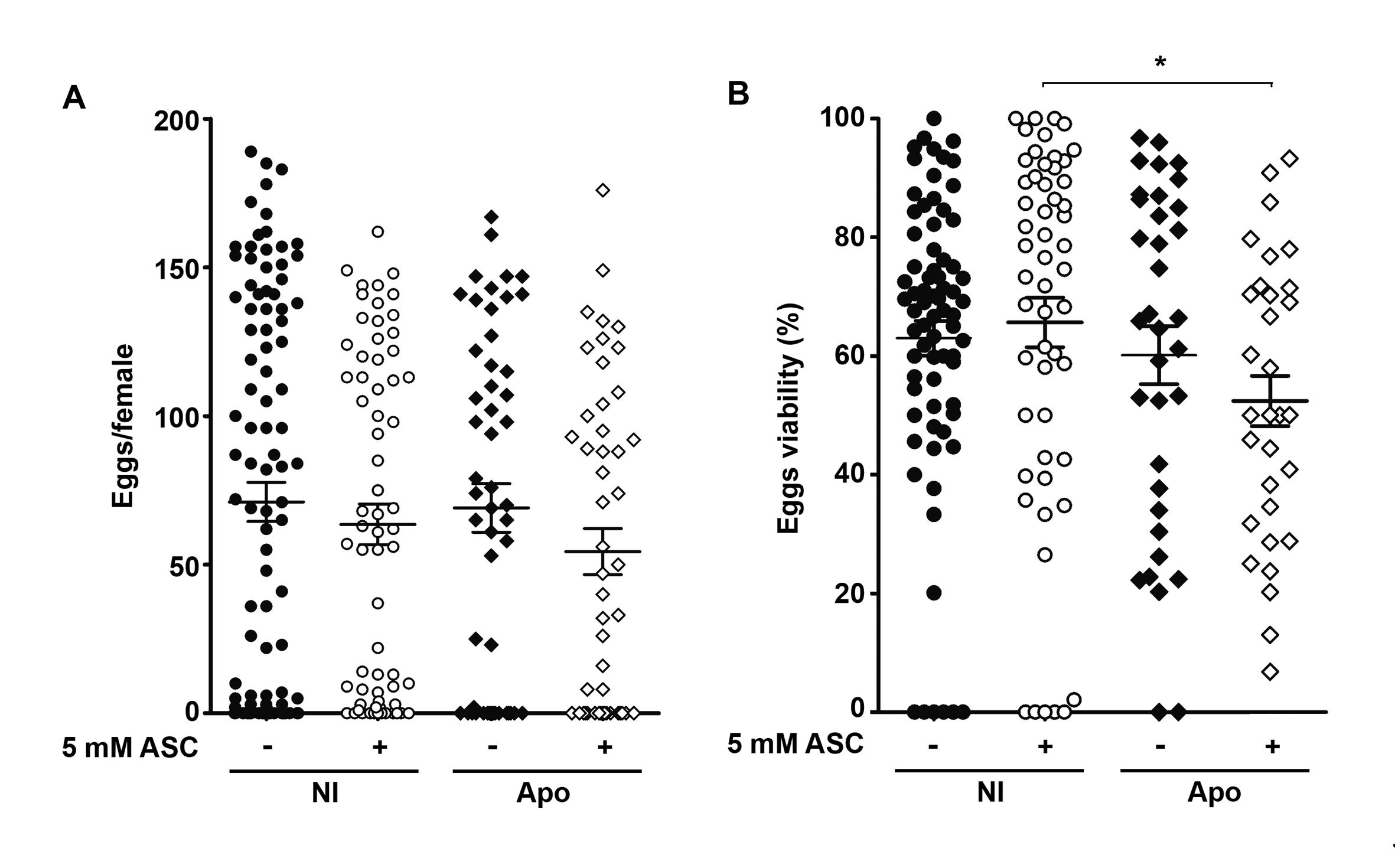

Supplement: Supplementary Figure 5 — Apo infection does not impair the reproductive fitness of A. aegypti females. (A) Egg production and (B) viability were evaluated in non-infected (NI) and Apo-infected females fed with 10% sucrose or 10% sucrose + 5 mM ASC ad libitum (*P = 0.01). Synchronized oviposition was stimulated 4 dpi. Significant p-values were obtained by the Mann-Whitney test (***P < 0.01) and error bars represent mean ± SEM of two independent experiments. [file Image_5.tif]
