# Supplementary material for: Aedes aegypti Infection With Trypanosomatid Strigomonas culicis Alters Midgut Redox Metabolism and Reduces Mosquito Reproductive Fitness
Source: Front Cell Infect Microbiol. 2021 Aug 13;11:732925. doi: 10.3389/fcimb.2021.732925 (PMC8414984; doi:10.3389/fcimb.2021.732925)
Supplement: Supplementary Table 1 — Oligonucleotides sequences used in qPCR assays. [file Table_1.docx]

**SUPPLEMENTARY MATERIAL**

**Table S1:** Oligonucleotides sequences used in qPCR assays

| **Gene** | **Gene ID** | **Oligonucleotidessequences (5’-3’)** | **References** |
| --- | --- | --- | --- |
| **TR** | 528246207 | AGAGCGGAAAGGAGGCGGAC *For* | (Bombaça et al., 2017) |
|  |  | CGCCGACCTTGTTGAGCTGC *Rev* |  |
| **cTXNPx** | 528243040 | CGCTAAGCTTAACCACCCCG *For* |  |
|  |  | TCTTCTTGAAGGTGCCGTCC *Rev* |  |
| **mTXNPx** | 528224473 | TGTGTGCCCGACCGAAATTA *For* |  |
|  |  | CGCCAAGTGTGAATACTGCG *Rev* |  |
| **GAPDH** | 528252126 | AGTGCGCATCTGACTCGTTT *For*  GGGTTGCGATTAGCAAGCAT *Rev* |  |
| ***cact*** | AAEL000709 | AGACAGCCGCACCTTCGATTCC *For* |  |
|  |  | CGCTTCGGTAGCCTCGTGGATC *Rev* | (Xi et al., 2008) |
| ***casp*** | AAEL003579 | GAATCCGAGCGAGCCGATGC *For* |  |
|  |  | CGTAGTCCAGCGTTGTGAGGTC *Rev* |  |
| ***Rel2*** | AAEL007624 | GCTCAGTGCTACCGTGGGAAAC *For* |  |
|  |  | CGGGTTCGCTCTGGCATTTGTC *Rev* |  |
| ***Att*** | AAEL003389 | TTGGCAGGCACGGAATGTCTTG *For* |  |
|  |  | TGTTGTCGGGACCGGGAAGTG *Rev* |  |
| ***DefC*** | AAEL003832 | TTGTTTGCTTCGTTGCTCTTT *For* |  |
|  |  | ATCTCCTACACCGAACCCACT *Rev* |  |
| ***DefA*** | AAEL003841 | CTGCCGGAGGAAACCTATCAG *For* | (Dong et al., 2012) |
|  |  | GCAATGCAATGAGCAGCACAAG *Rev* |  |
| **Ribosomal protein S7** | AAEL009496 | GGGACAAATCGGCCAGGCTATC *For* |  |
|  |  | TCGTGGACGCTTCTGCTTGTTG *Rev* |  |
| ***dome*** | AAEL012471 | AAACGGTGGCAAAATGAACT *For* | (Souza-Neto et al., 2009) |
|  |  | CATACAGCCGGCTTTCTTCT *Rev* |  |

**Supplementary Figure 1:** Apo infection persistence in ASC-fed females increases ROS-dependent response. Analysis was performed in midguts of non-infected (NI) and Apo-infected females fed with 10% sucrose or 10% sucrose + 5 mM ASC *ad libitum* at 1 and 4 dpi (*P = 0.01, **P = 0.005, ***P = 0.01). Quantitative analysis of fluorescence microscopy was performed individually in 10-15 midguts per group. Significant P values were obtained by Mann-Whitney test and error bars represent mean ± SEM of three independent experiments.

**Supplementary Figure 2:** DPI feeding decreased DHE staining in *S. culicis* infected midguts. Analysis was performed in midguts of non-infected (NI), WT- and WTR-infected females fed with 10% sucrose or 10% sucrose + 10 µM DPI *ad libitum* 4 dpi. (A) DUOX activity of NI in response to incubation with 1 µM ionomycin, 5 µM DPI or 5 mM EGTA (*P = 0.05). Enzyme activity was performed through the production of H_2_O_2_ in pools of 20 guts. (B) Representative confocal images of midguts dissected and incubated with DHE. Scale bar represents 20 µm. Arrowheads indicate high staining in WTR-infected midgut. Representative images of three independent experiments. Significant P values were obtained by MannWhitney test and error bars represent mean ± SEM of at least three independent experiments.

**Supplementary Figure 3:** *S. culicis* infects Aag2 cell culture by flagellum insertion in epithelial cells. Scanning electron microscopy analysis of (A, C, D) WT and (B,D,F) WTR interaction with Aag2 at 4 h. Arrowheads point adhered parasites, being possible to observe similar infection rates between WT and WTR infections. Parasite-host interaction also occurs similarly in both infections, mainly by flagellum insertion (arrows). Scale bars represent 10 µm (A–D) and 5 µm (E, F). Representative micrographs of three independent experiments.

**Supplementary Figure 4:** Menadione treatment impairs WT infection in Aag2 cell cultures after 4 h. Epithelial cells were treated with 0.25 µM DPI, 50 U/mL catalase, 5 µM menadione, 1 µM mitoTEMPO or 50 U/mL SOD for 2 h before the infection. After that, the infection with WT and WTR strains was performed for 4 h. Non-adhered parasites were discarded and Aag2 cell culture fixed to infection evaluation or maintained until 24 h. Percentage of infected cells at (A, D) 4 or (C) 24 h (*P = 0.01). (B, E) Number of adhered parasites per 100 cells. Significant P values were obtained by Mann-Whitney test and error bars represent mean ± SD of at least three independent experiments.

**Supplementary Figure 5:** Apo infection does not impair the reproductive fitness of *A. aegypti* females. (A) Egg production and (B) viability were evaluated in non-infected (NI) and Apo-infected females fed with 10% sucrose or 10% sucrose + 5 mM ASC ad libitum (*P = 0.01). Synchronized oviposition was stimulated 4 dpi. Significant P values were obtained by the Mann-Whitney test (***P < 0.01) and error bars represent mean ± SEM of two independent experiments.

**REFERENCES**

Bombaça, A.C.S., Dias, F. de A., Ennes-Vidal, V., Garcia-Gomes, A. dos S., Sorgine, M.H.F., d’Avila-Levy, C.M., et al. (2017). Hydrogen Peroxide Resistance in *Strigomonas culicis*: Effects on Mitochondrial Functionality and *Aedes aegypti* Interaction. Free Radic. Biol. Med. 113:255-266. doi: 10.1016/j.freeradbiomed.2017.10.006.

Xi, Z., Ramirez, J.L., and Dimopoulos, G. (2008). The *Aedes aegypti* Toll Pathway Controls Dengue Virus Infection. PLoS Pathog. 4(7):e1000098. doi: 10.1371/journal.ppat.1000098.

Dong, Y., Morton, J.C., Ramirez, J.L., Souza-Neto, J.A., and Dimopoulos, G. (2012). The Entomopathogenic Fungus Beauveria Bassiana Activate Toll and JAK-STAT Pathway-Controlled Effector Genes and Anti-Dengue Activity in *Aedes aegypti*. Insect Biochem. Mol. Biol. 42(2):126-132. doi: 10.1016/j.ibmb.2011.11.005.

Souza-Neto, J.A., Sim, S., and Dimopoulos, G. (2009). An Evolutionary Conserved Function of the JAK-STAT Pathway in Anti-Dengue Defense. Proc. Natl. Acad. Sci. U. S. A. 106(42):17841-17846. doi: 10.1073/pnas.0905006106.
